# Natural Born Laser Dyes: Excited-State Intramolecular Proton Transfer (ESIPT) Emitters and Their Use in Random Lasing Studies

**DOI:** 10.3390/nano9081093

**Published:** 2019-07-30

**Authors:** Julien Massue, Thibault Pariat, Pauline M. Vérité, Denis Jacquemin, Martyna Durko, Tarek Chtouki, Lech Sznitko, Jaroslaw Mysliwiec, Gilles Ulrich

**Affiliations:** 1 Institut de Chimie et Procédés pour l’Energie, l’Environnement et la Santé (ICPEES), UMR CNRS 7515, Ecole Européenne de Chimie, Polymères et Matériaux (ECPM), 25 Rue Becquerel, CEDEX 02, 67087 Strasbourg, France; 2 CEISAM, UMR CNRS 6230, BP 92208, 2 rue de la Houssinière, CEDEX 03, 44322 Nantes, France; 3 Advanced Materials Engineering and Modeling Group, Wroclaw University of Science and Technology, Wybrzeze Wyspianskiego 27, 50-370 Wroclaw, Poland

**Keywords:** ESIPT, random lasing, fluorescence, ab initio calculations, excited states

## Abstract

A series of five excited-state intramolecular proton transfer (ESIPT) emitters based on a 2-(2′-hydroxyphenyl) benzoxazole (HBO) scaffold, functionalized with a mono-or bis-(trialkylsilyl) acetylene extended spacer are presented. Investigation of their photophysical properties in solution and in the solid-state in different matrix, along with ab initio calculations gave useful insights into their optical behavior. Random lasing studies were conducted on a series of PMMA doped thin films, showing the presence of stimulated emission above the threshold of pumping energy density (ρth ≈ 0.5–2.6 mJ cm^−2^). In this work, the similarity of four level laser systems is discussed in light of the ESIPT photocycle.

## 1. Introduction

Excited-state intramolecular proton transfer (ESIPT) single or dual emitters serve as efficient fluorophores owing to their attractive optical properties, including large Stokes’ shifts, strong solid-state luminescence efficiency and environment-sensitive emission bands ratio [1,2,3,4,5]. Optimized ESIPT dyes have been successfully applied in a wide array of fields, spanning from optoelectronics [6,7] to cellular fluorescence mapping [8,9], multiple stimuli sensing [10] and polymer science [11]. Although several families of ESIPT dyes have been developed, including compounds derived from imidazole [12,13], pyrazole [14], hydroxytriazine [15] or 2,2′-bipyridine-diol [16] cores, ESIPT fluorophores incorporating a 2-(2′-hydroxyphenyl) benzazole (HBX) scaffold remain one of the most interesting families due to their versatile π-conjugated structure, expedite synthesis and overall beneficial modularity [17,18,19,20]. ESIPT emitters generally undergo numerous molecular vibrations, motions or rotations in solution leading to the presence of non-radiative deactivation pathways detrimental for the emission quantum yield [21]. Notably, following proton transfer, a twist between the two moieties of the dye can take place, leading ultimately to a conical intersection (CI) with the ground state, and hence, an effective non-radiative decay. In the solid-state, however, these detrimental motions are usually restricted or hindered and an enhancement of the fluorescence intensity is generally observed. Recently, several groups have successfully developed ESIPT emitters displaying good photoluminescent quantum yield in solution and in the solid-state, enlarging the potential fields of applications for these innovative dyes [22,23,24]. Nevertheless, providing additional examples of ESIPT dyes displaying strong fluorescence in solution and in the solid-state is still of high interest. Indeed, the vast majority of dyes is usually fluorescent only in solution but faintly in the solid-state due to multiple aggregation-caused quenching (ACQ) processes. In this framework, ESIPT dyes can be viewed as very valuable members of the aggregation-induced emission (AIE) or aggregation-induced enhanced emission (AIEE) dye families that are poorly luminescent in solution owing to the presence of numerous molecular rotors but strongly brighten up in the solid-state when these rotations are restricted [25,26]. Efficient solid-state emitters find significant applications including security printing, optoelectronics and amplified stimulated emission (ASE) devices [27]. Among ASE dyes, very few are based on an ESIPT process and are all studied in the crystalline state [28,29,30,31,32,33] which can be tricky to obtain, since the presence of alkyl groups on the molecular core, usually required to ensure solubility during the synthesis, can impede the crystallization processes. The unique and peculiar spectral properties of ESIPT dyes make them ideal candidates for studies on light enhancement phenomena such as random lasing (RL) [6,7,27]. The term random laser (RL) refers to a non-conventional laser based on all physical systems that use disorder and multiple constructive light scattering in the light amplifying medium to supply positive feedback for laser operation, as opposed to the reflective feedback by the mirrors of the conventional laser [34,35]. RL is connected to optical scattering in the active medium strong enough to play a positive role in light amplification, as predicted first in 1968 [36]. A conventional laser is based on an optically active medium that amplifies light by stimulated emission enclosed by a cavity, typically a pair of mirrors, facing each other determining the modes of a laser, which are the frequency and directionality of the output. Lasing occurs only when the total gain in the cavity overcomes the losses. Coherent amplification is achieved by maintaining the active medium in a state of population inversion through a pumping mechanism. RL works on the same basic principles but the light propagation is more or less similar to that of the Brownian movements of the particles suspended in a liquid [34]. Even though multiple scattering is a reported and established phenomenon, the RL process is still not completely understood due to the numerous interference effects. Nevertheless, low fabrication cost, small size, flexible shape, and substrate compatibility are some of the many advantages that justify RL to be used in a wide array of applications such as search-and-rescue identifications, photodynamic therapy or as a miniature light source in integrated photonic circuits [37]. In recent years a great deal of attention has been put to new systems for light amplification, based on a wide range of materials: spanning from metal-based and hybrid networks, through conjugated polymers and organic dyes, to biomaterials. All of these systems strive to lower the lasing threshold, which is directly related to the luminescent properties of a given material. Light self-absorption can be very detrimental for efficient RL which makes ESIPT dyes with their intrinsic large Stokes shift very attractive compared to previously reported RL materials [38,39,40].

**Figure 1:**
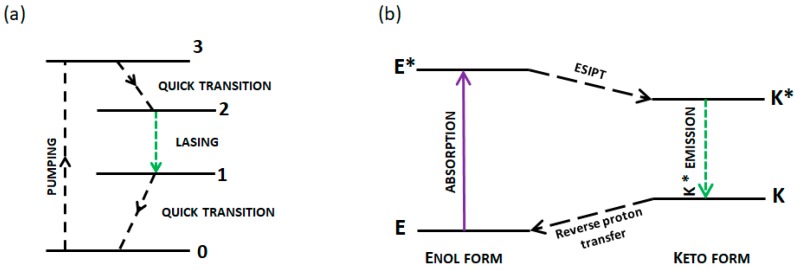
Energy diagrams of (**a**) the four-level laser system and (**b**) the excited-state intramolecular proton transfer (ESIPT) photocycle.

Both random and conventional lasers can be divided into three- and four-level systems, based on the number of energy states involved in the light amplification process. Typically laser dyes represent a four-level system where the rich vibrionic structure of excited and ground states of a dye is involved in constitution of laser emission. That requires a developed π-conjugated system as a part of the dye structure. In case of ESIPT type molecules, a four-level system can be formed utilizing electronic states appearing as a consequence of the ESIPT photo-cycle (E–E*–K*–K) (see Figure 1). In such cases, the reservoir electrons of the dye are pumped directly to the third level (E*), from where they relax almost immediately to the second level (K*). In this way, a population inversion between the second (K*) and the first level (K) is reached, as the K tautomer is much less stable than E and hence has nearly zero population at the equilibrium. Therefore, the electrons relax coherently into the first level via stimulated emission, which is essentially the process of lasing. Finally, the electrons relax to the ground level (E). The transition from level 1 to level 0 must be very fast (e.g., through non-radiative processes) for the radiation obtained in such a laser to be continuous, and this should be the case in ESIPT dyes, as the transition-state between K and E is usually trifling. In the case of three-level systems, the first level is empty which means that each atom excited to level 2 causes population inversion. Therefore, a four-level system may be more advantageous than a three-level system, in terms of efficiency which could be however limited by the higher energy of the quantum defect (which is negated in RL). In the case of the ESIPT emitters studied here, the similarity between the described four-level process and the ESIPT photocycle is evident, as the ESIPT process and reverse proton transfer could be compared to quick transition between levels 3 and 2 and between levels 1 and 0, respectively.

**Figure 2:**
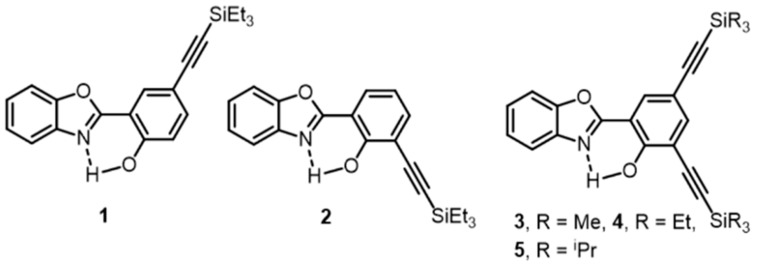
Molecular structures of the studied 2-(2′-hydroxyphenyl) benzoxazole (HBO) fluorophores.

The structure of the 2-(2′-hydroxyphenyl) benzoxazole (HBO)-based ESIPT studied here is given in Figure 2. They featured mono-or bis-ethynyl extended trialkylsilyl moieties as these spacers have been reported to significantly enhance the photoluminescent quantum yield both in solution and in the solid-state [24]. The position of the alkynyl functionalization, as well as the nature of the alkyl group were modified in order to observe the influence of small structural modifications on the optical properties and RL efficiency.

## 2. Materials

HBO dyes **3** and **4** were synthesized according to reported procedures [24,41]. Generally speaking, HBO dyes **1**–**5** were prepared using a straightforward two-step procedure starting from commercially available mono-or bis-halogenated salicylaldehyde and 2-aminophenol. The resulting HBO heterocycles intermediates were then involved in a Pd-catalyzed Sonogashira cross-coupling reaction with an excess of trialkylsilylacetylene in toluene in the presence of 5% catalyst. Pure HBO **1**–**5** were obtained as white powders after purification on a column chromatography. These dyes were found to be highly soluble in a large range of solvents owing to the beneficial presence of alkyl groups. It is noteworthy that HBO dyes **1**–**5** can be easily obtained on a multi-gram scale owing to their high-yielded two-steps synthesis. All new compounds were characterized by ^1^H and ^13^C NMR spectroscopy and high-resolution mass spectrometry (HR-MS, micrOTOF II, Bruker, USA). All spectra can be found in the Supplementary Material.

All experimental details can be found in the Supplementary Material. 

## 3. Results and Discussion

### 3.1. Photophysical Properties

**Figure 3:**
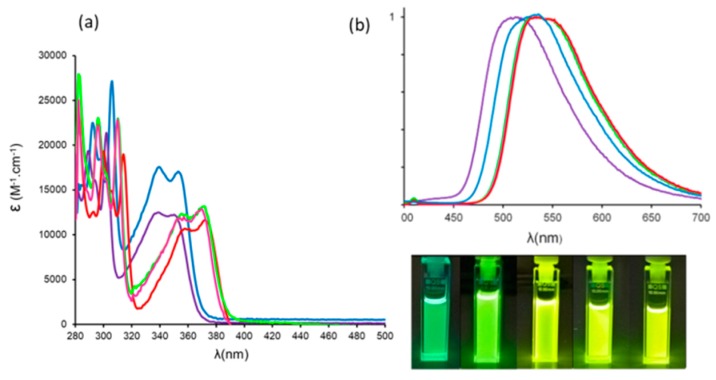
(**a**) UV-Visible spectra of HBO dyes **1** (purple), **2** (blue), **3** (red), **4** (green) and **5** (pink) recorded in toluene in aerated solution at 25 °C, (**b**) emission spectra of HBO dyes **1** (purple), **2** (blue), **3** (red), **4** (green) and **5** (pink) recorded in toluene in aerated solution at 25 °C, (**c**) photographs of toluene solutions of HBO **1**–**5** (from left to right) under irradiation at λ_exc_ = 365 nm.

The photophysical data recorded in toluene and ethanol for HBO dyes **1**–**5** are listed in Table 1. The spectra for HBO dyes **1**–**5** in toluene at 25 °C are presented on Figure 3.

As a general trend, HBO fluorophores **1**–**5**, functionalized with one or two π-extended trialkylsilylalkynyl moieties at positions 3 and/or 5 of the phenol cycle displayed absorption spectra of rather similar shape. A relatively structured absorption band located in the UV part of the electromagnetic spectrum (λ_abs_ = 335–371 nm) was observed in each dye (Figure 3). The molar absorption coefficients were in the 8600–17,000 M^−1^·cm^−1^ range which were expected values for such compact heterocycles.

The absorption wavelengths of mono-substituted HBO dyes **1** and **2** displayed small hypsochromic shifts compared to their bis-substituted analogues **3**–**5** (λ_abs_ = 337–345 nm for HBO **1**–**2** vs. λ_abs_ = 367–371 nm for HBO **3**–**5**). The absorption spectra for dyes **1**–**5** in ethanol can be found in the Supplementary Material. These observations highlight the fact that a second trialkylsilylalkynyl unit is necessary to induce a red-shifted absorption regardless of the position of the first unit on the π-conjugated molecular core. Among the bis-substituted series, no drastic change is observed in terms of shape or position of the absorption band upon increasing the length of the alkyl group (Me, Et or iPr for HBO **3**–**5** respectively). Additional bands below 300 nm can also be observed on the absorption spectra and assigned to the π–π* transitions of the aromatic cycles.

In toluene, irradiation in the lowest energy absorption band (λ_exc_ = 330–370 nm) led to the appearance of an intense single emission band which was assigned to the decay of the keto tautomer formed in the excited state (K*), as a result of the ESIPT process (Figure 3b). In aprotic toluene, ESIPT appeared to be a quantitative process in all cases, leading to red-shifted single emitters with Stokes’ shifts higher than 8000 cm^−1^. This was confirmed by theoretical calculations (vide infra). In protic ethanol, a second emission band was observed at higher energies for HBO dyes **1** and **4**, which was assigned to the radiative decay from the enol tautomer (E*) in its excited state (see the Supplementary Materials), evidencing a partial frustration of the ESIPT process. Protic solvents tend to stabilize the enol tautomer in the excited states, as reported for many ESIPT probes [1,2,3,4,5].

**Figure 4:**
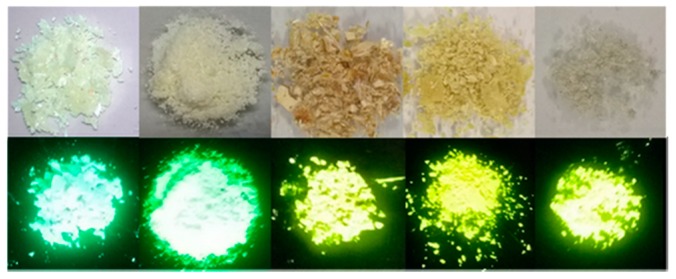
Photographs of HBO **1**–**5** (from left to right) as powders under daylight (**top**) and under irradiation at λ_exc_ = 365 nm (**bottom**).

**Figure 5:**
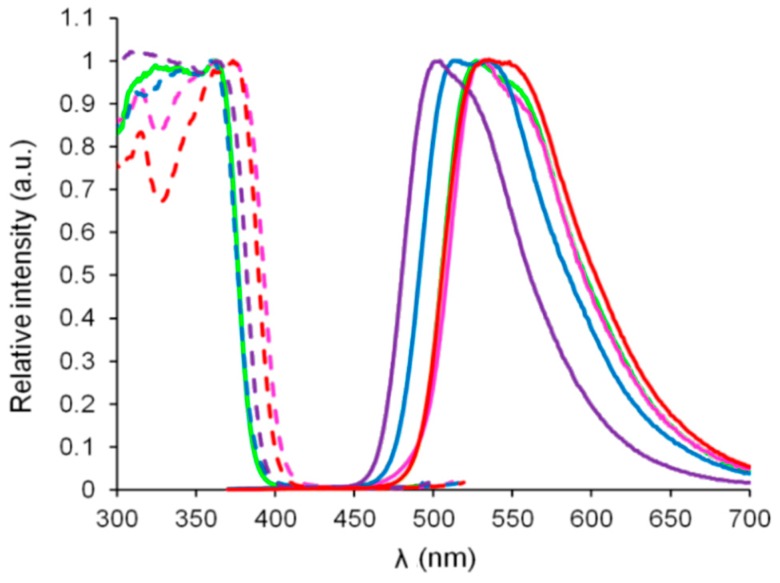
Excitation (dotted line) and emission (plain lines) spectra of HBO dyes **1** (purple), **2** (blue), **3** (red), **4** (green), and **5** (pink) in the solid-state as embedded in KBr pellets (concentration around 10^−6^ M).

**Figure 6:**
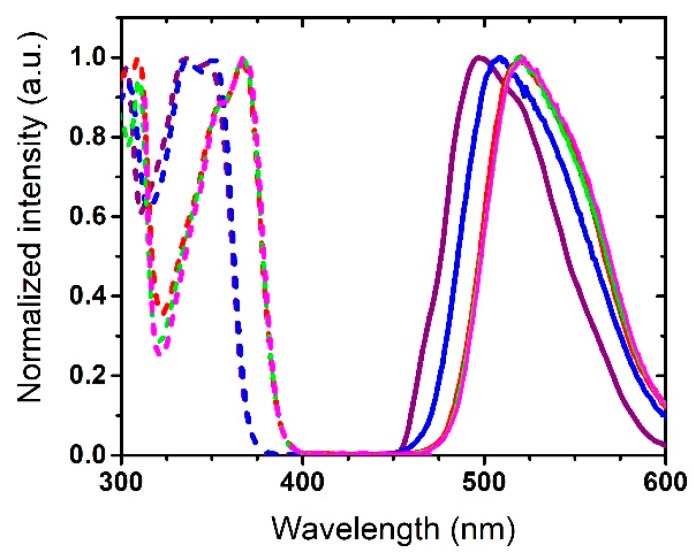
Excitation (dotted line) and emission (plain lines) spectra of HBO dyes **1** (purple), **2** (blue), **3** (red), **4** (green), and **5** (pink) in the solid-state as doped at 1% in PMMA films.

We underline that all emission bands were located in the visible region for HBO dyes **1**–**5** (λ_em_ = 513–539 nm in toluene, see Table 1). HBO fluorophores **1** and **2**, functionalized with a single triethylsilylalkynyl unit in the 5 or 3 positions of the phenol cycle, displayed maximum emission wavelengths at 513 and 538 nm in toluene respectively. Functionalization in the ortho position of the hydroxy group as in HBO **2**, led to a sizable red-shift of the emission, as compared to the para substitution (HBO **1**). The fluorescence quantum yields in toluene were 11% and 28% for **1** and **2**, respectively. These values were significantly higher than those usually obtained for simple HBO scaffolds in similar conditions. This was especially true for HBO **2** where the presence of an ethynyl segment adjacent to the H-bond donor led to an important increase of the radiative constant as compared to HBO 1 (k_r_ = 0.28 × 10^8^ s^−1^ vs. k_r_ = 0.97 × 10^8^ s^−1^ for HBO **1** and **2** respectively). The introduction of a second trialkylsilylalkynyl moiety onto the HBO scaffold triggered only a slight bathochromic shift of the emission as compared to mono-substituted dye **1** but yielded comparable emission wavelengths as **2** (λ_em_ = 537–539 nm in toluene for HBO **3**–**5**). The fluorescence lifetimes were in the nanosecond timescale which were expected values for organic singlet emitters. However, the fluorescence quantum yield appeared to be significantly enhanced for HBO **3**–**5** peaking at 49% in toluene for HBO **3**. The quantum yields remained high in the double substituted HBO series **3**–**5** (Φ = 32–49%) with a slight drop upon increase of the length of the silylalkyl group; a feature explained by the vibrations triggered by the flexible unsaturated arms. The differences of quantum yields between HBO **1**, **2**, and **3**–**5** were further rationalized by theory (vide infra). It is also interesting to emphasize HBO emitters **3**–**5** remained fluorescent in ethanol with fluorescence quantum yields spanning from 28% to 33%. These observations bring insightful feedback for the design of future ESIPT probes. HBO **1**–**5** showed bright luminescence to the naked eye as amorphous powders under irradiation with a standard 365 nm bench UV lamp (Figure 4). This prompted us to investigate their optical properties in the solid-state as dispersed in a potassium bromide (KBr) matrix or doped as 1% in poly (methyl methacrylate) (PMMA) films. The photophysical properties of ESIPT emitters **1**–**5** in the solid-state are presented in Table 2 while the excitation and emission profiles recorded in KBr pellets and PMMA films are displayed in Figure 5 and Figure 6 respectively.

The excitation and emission spectra of HBO **1**–**5** recorded in KBr pellets adopted similar shapes and maximal emission wavelengths as in toluene, i.e., an intense, poorly structured single K* band located in the green–yellow region (λ_em_ = 504–534 nm) (Figure 5). Encapsulation of these ESIPT emitters in a confined matrix led to a significant enhancement of the fluorescence quantum yields, which were calculated to range from 53% to 82%. In doped PMMA films, a single emission profile, corresponding to the decay of K* band was observed for all dyes. The emission wavelengths spanned from 490–521 nm (Figure 6). This photophysical behavior indicated a strong dependence of the emission profile on the vicinal environment of the dye, a feature frequently encountered for ESIPT probes. It is noteworthy that the quantum yields in PMMA films were significantly lower than in KBr pellets (Φ = 20–49%).

### 3.2. Ab Initio Calculations

**Figure 7:**
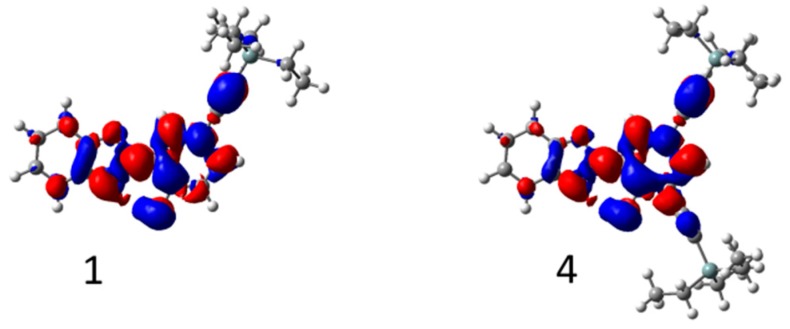
Density difference plot corresponding to electronic excitation of HBO **1** and **4**. The blue and red regions respectively correspond to decrease and increase of electronic density upon absorption. See S6, Scheme S6.2 in the Supplementary Material for the other dyes.

First-principle methods were used to probe the nature of the electronic excited states (see the Supplementary Materials for details). Key results are summarized in Table 3. For the absorption, theory returned absorption maxima blue-shifted compared to experimental results, which was ascribed to the lack of vibrionic coupling in our calculations. More importantly, the bathochromic shift of ca. 25 nm noticed experimentally when going from HBO **1**–**2** to HBO **3**–**5** (Table 1) was nicely reproduced by the calculations. In Figure 7, electron density difference plots obtained for ESIPT emitters **1** and **4** are presented (see the Supplementary Materials for the full data). They show that the ethynyl segments were involved in the excitation process, explaining why the presence of two trialkylsilyl moieties in HBO **3** and **5**, logically yielded to bathochromic shifts, as compared to the monosubstituted dyes **1** and **2**. The alkyl groups did not play any direct role in the excitations, which could explain why the absorption bands of HBO **3**–**5** were very similar. Upon absorption of a photon, the hydroxy group became more acidic (decrease of electron density) whereas the nitrogen atom became more basic (gain of electronic density), which is an excited state topology favorable for ESIPT (Figure 7). The emission wavelengths determined by the calculations for K* (Table 3) were in very good agreement with their experimental counterparts (Table 1), confirming that the emissions were solely originating from the keto tautomers in toluene. Specifically, the redshift observed between the emissions of HBO **1** and **2**, absent in the absorption values, was nicely reproduced by theory. More importantly, the computed driving forces for proton transfer (ΔG^K^*^–E^*) were all strongly negative and the associated ESIPT barriers (ΔG^TS^*) were also negative. The transition-state was therefore below the enol form on the free energy scale (it was of course above on the total energy scale), which was indicative of an unstable E* form and a barrierless proton transfer process, consistent with quantitative ESIPT [42]. This explains the absence of experimental E* emission in toluene. Experimentally, the emission quantum yield decreased when longer alkyl group were used, i.e., when going from HBO **3** to HBO **5**, which was, as stated above, the expected consequence of additional vibrational degrees of freedom. More importantly, there were also significant differences of quantum yields between HBO **1**–**5.** As ESIPT is quantitative, the most well-known deactivation mechanism from K*, that is, the twisting around the central bond separating the two rings in the K* form, has also been investigated (see S6 in the Supplementary Materials) [43,44]. The higher the twisting barrier (T^S2^*) is, the less likely the system is to go back to the ground state through a conical intersection, and the larger the emission quantum yield. As can be seen in Table 3, a low barrier was computed for HBO **1** (0.06 eV), indicating that this deactivation path should be rather efficient in that dye, and significantly higher barriers were determined for the dyes containing an ethynyl segment at the ortho position (>0.10 eV), which was consistent with the experimental trends of a relatively small emission quantum yield for HBO **1** and significantly higher responses for dyes **2**–**5**.

### 3.3. Random Lasing Studies

**Figure 8:**
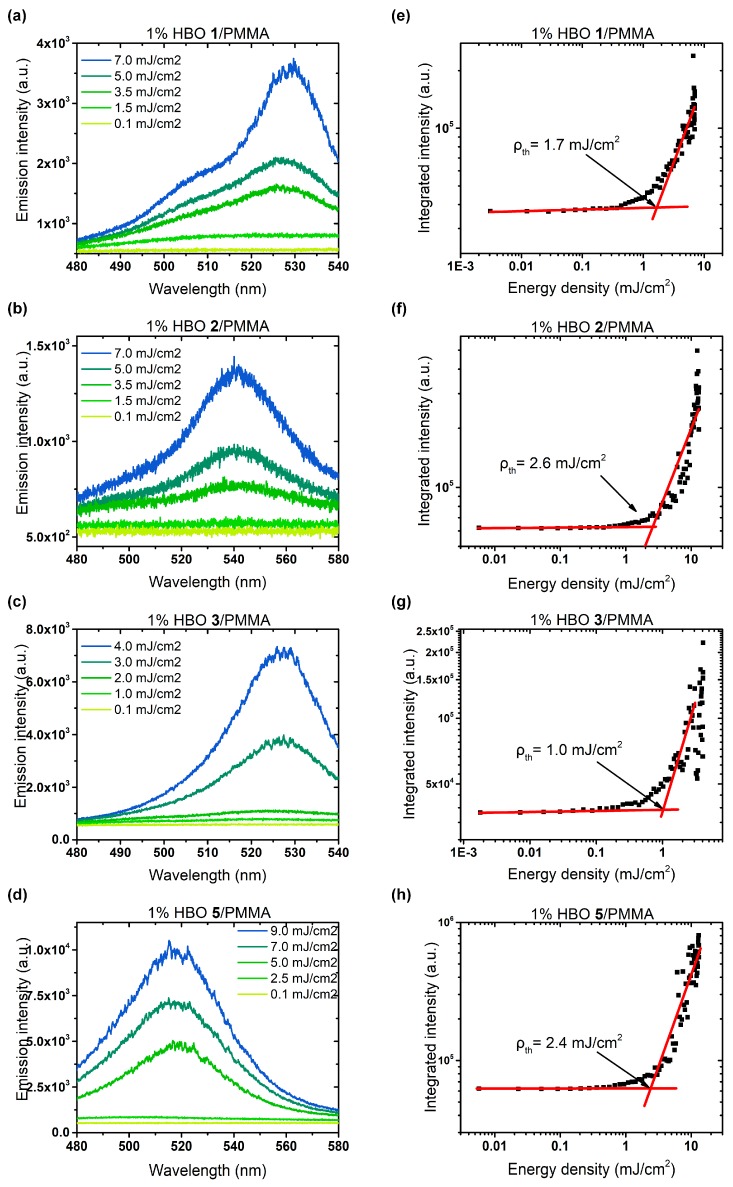
(**a**–**d**) Dependence of the light emission spectra on the excitation energy density for 1% doping of ESIPT dyes **1**–**3** and **5** in the PMMA sample and (**e**–**h**) corresponding integrated emission intensity as a function of the pumping energy density for the same samples, along with the calculated threshold.

The RL studies were performed for samples with varying dye loadings (see the Supplementary Materials for details). Both coherent and incoherent RL was observed for all studied ESIPT dyes. With the increase of the pumping energy density, the gain started to overcome the losses, which resulted in an increase of the intensity along with the narrowing of the emission profile. Representative spectra of RL for HBO dyes **1**–**3** and **5** are given in Figure 8a–d (and S7, Figure S7.4), while the integrated emission intensity dependence on the exciting energy density is presented in the log–log scale in Figure 8e–h. The RL studies on HBO **4** have been reported elsewhere [45]. The stimulated emission occurs above the threshold level of pumping energy density, as evidenced by a rapid increase of the emission intensity.

**Figure 9:**
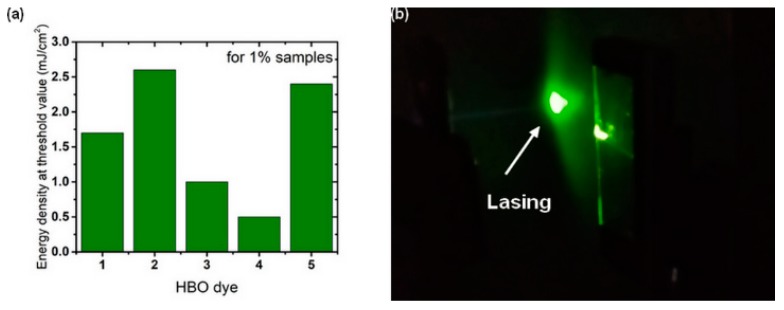
(**a**) Difference between random lasing threshold values for 1% samples of HBO dyes **1**–**5** in PMMA matrix and (**b**) a photography of the stimulated emission above the RL threshold observed for 1% doped HBO **5** in the PMMA sample.

In Figure 9a, the difference in RL threshold values for the samples with 1% of dyes is presented. By calculation, the threshold of pumping energy density (ρth) was estimated to be varying between 0.5 to 2.6 mJ cm^−2^, which was on a par with typical threshold values recorded for organic fluorophores incorporated in polymer matrix. Moreover, the RL emission did not appear to be affected by concentration-related quenching effects.

The maximum emission wavelength for all dyes was centered at ca. 525–555 nm and could be clearly correlated to the emission originating from the excited keto (K*) band in the light intensity enhancement. Additionally, for HBO dyes **1** and **2**, the presence of another band could be observed, appearing only for the 5% doped polymeric thin films, even though such phenomena was not observed during the fluorescence measurements (see S7, Figures S7.1–S7.3). For HBO **1** and HBO **2**, a blue-shifted emission influenced by an increase in the dye concentration was also observed (see the Supplementary Materials). Furthermore, functionalization with a mono triethylsilylalkynyl unit at the ortho position of the phenol cycle, as in HBO **2** induced a two-fold increase in RL threshold value, as compared to a para substitution (HBO **1**), going from 1.7 mJ cm^−2^ to 2.6 mJ cm^−2^ (Figure 9a).

Appearance of high and narrow (<0.3 nm) spikes in the emission spectra after threshold was observed for all samples with higher concentration of the dye in polymer matrix (see S7, Figure S7.4). Such profiles are typical for coherent random lasing. Also, the differences in the threshold values, related both to the structure and concentration, can be explained by a change in scattering mechanisms involved in the formation of a positive feedback loop for RL emission. For lower HBO concentrations (ca. 3%), the spontaneously formed defects and layer irregularities formed during polymer drop casting resulted in light scattering, while the gain was provided by the molecular emission coming from the dye itself. Slowly ongoing crystallization and aggregation occurred at higher concentrations, thus increasing the restriction of intramolecular rotation of the molecules, giving rise to an additional feedback from light scattering on the crystals. The decrease in the non-radiative processes ultimately results in a lowering of the threshold (see S7, Figure S7.5).

At 5% doping for HBO **1**/PMMA and HBO **2**/PMMA samples, it seemed clear that the light enhancement originated from both the excited keto states (K*) and aggregates, still working as a four-level system. Moreover, the analysis of Figure 9a made us hypothesize that the substitution with triethylsilylalkynyl unit in both 5 and 3 positions of the phenol ring (HBO **4**) had a strong influence on the RL mechanism, by lowering the threshold value.

Additionally, photodegradation studies were conducted. A general increase in photostability could be observed alongside higher amounts of doping in polymer matrix. Results can be found in the Supplementary Material (see S7, Figure S7.6).

## 4. Conclusions

A series of original ESIPT emitters based on an HBO molecular scaffold was synthesized and studied as novel organic fluorophores for RL. These dyes present a mono-or a bis-ethynyl-extended trialkylsilyl substitution on the phenol ring. The study of their optical properties in solution showed the presence of a single K* emission band in toluene with an enhanced photoluminescent quantum yield as compared to unsubstituted analogues. These dyes appeared to be also very fluorescent in the solid-state, incorporated in KBr pellets or doped in PMMA films. These observations were rationalized using ab initio calculations.

Additionally, these dyes were studied as potential RL emitters. RL, both coherent and incoherent, was observed for all dyes with threshold values that could be fine-tuned depending on several parameters such as the number of alkynyl moieties on the molecular core and the nature of the alkyl groups. Taking advantage of the similarity between the four-level process required for a laser and the ESIPT photocycle, these reported ESIPT fluorophores can be viewed as natural born laser dyes, paving the way to the engineering of innovative applications.

## Figures and Tables

**Table 1:** Photophysical data of HBO dyes **1**–**5** measured in aerated solution at 25 °C.

Dye	λ_abs_ (nm)	ε (M^−1^·cm^−1^)	λ_em_ (nm)	ΔSS (cm^−1^)	Φ_F_^[a]^	τ (ns)	k_r_ (10^8^ s^−1^) ^[b]^	k_nr_ (10^8^ s^−1^) ^[b]^	Solvent
1	345	11,900	513	9500	0.11	3.9	0.28	2.28	Toluene
1	339	11,700	407/504	4900	0.05	2.7	0.18	3.52	EtOH
2	340	17,000	538	10,800	0.28	2.9	0.97	2.48	Toluene
2	337	16,400	524	10,100	0.19	1.9	1.00	4.26	EtOH
3	368	12,800	539	8600	0.49	3.9	1.26	1.31	Toluene
3	367	8600	524	8200	0.33	3.0	1.10	2.23	EtOH
4	371	15,400	537	8300	0.38	3.7	1.41	2.30	Toluene
4	368	14,000	459/524	5400	0.28	3.2	0.88	2.25	EtOH
5	371	13,800	535	8300	0.32	3.8	0.84	1.79	Toluene
5	368	12,500	526	8200	0.31	3.0	1.03	2.30	EtOH

^[a]^ Photoluminescent quantum yield determined in solution by using Rhodamine 6G as a reference (λ_exc_ = 488 nm, Φ = 0.88 in ethanol). ^[b]^ k_r_ (10^8^ s^−1^) and k_nr_ (10^8^ s^−1^) were calculated using the following equations: k_r_ = Φ_F_/τ, k_nr_ = (1 − Φ_F_)/τ.

**Table 2:** Photophysical data of HBO dyes **1**–**5** measured the solid-state.

	λ_exc_ (nm)	λ_em_ (nm)	ΔSS (cm^−1^)	Φf ^[a]^	Matrix
1	363	504	7600	0.66	KBr
1	337	430/516	5500	0.20	PMMA
2	359	514	8200	0.53	KBr
2	337	440/515	5800	0.22	PMMA
3	373	534	7800	0.70	KBr
3	363	450/526	5100	0.20	PMMA
4	363	527	7700	0.76	KBr
4	365	434/525	4200	0.49	PMMA
5	372	530	7900	0.82	KBr
5	364	457/527	5300	0.21	PMMA

^[a]^ Absolute quantum yields determined using an integration sphere.

**Table 3:** Computational results for HBO dyes **1**–**5** in toluene. The vertical absorption wavelength of the enol form and the vertical emission wavelength of the keto forms are reported as well as the difference of free energies on the excited-state potential energy surface (in eV).

	λ_abs_ (E) (nm)	λ_em_ (K*) (nm)	ΔG^K^*^–E^* (eV)	ΔG^TS^* (eV)	ΔG^TS2^* (eV)
1	317	510	−0.25	−0.15	0.06
2	317	532	−0.28	−0.10	0.12
3	332	547	−0.26	−0.07	0.16
4	334	550	−0.26	−0.11	^[a]^
5	335	552	−0.28	−0.09	0.17

^[a]^ The transition-state optimization did not converge to a physically meaningful structure. ΔG^K*–E*^ is the relative stability of the keto form with respect to the enol form in the excited state; ΔG^TS^* is the computed ESIPT barrier, that is the difference between the free energies of the proton transfer transition-state and the enol form; ΔG^TS2^* is the computed barrier between the keto form and the twisting conical intersection. See S6, Scheme S6.1 in the Supplementary Material for representation.

## Supplementary Materials

The following are available online at https://www.mdpi.com/2079-4991/9/8/1093/s1. S1: Synthetic schemes; S2. Experimental procedures; S3: ^1^H and ^13^C NMR traces; S4: HR-MS spectra; S5: Spectroscopic data; S6: Theoretical calculations; S7: Sample preparation for RL studies.
