# Supplementary material for: Natural Born Laser Dyes: Excited-State Intramolecular Proton Transfer (ESIPT) Emitters and Their Use in Random Lasing Studies
Source: Nanomaterials (Basel). 2019 Jul 30;9(8):1093. doi: 10.3390/nano9081093 (PMC6723810; doi:10.3390/nano9081093)
Supplement: Supplementary file 1 [file nanomaterials-09-01093-s001.pdf]

Electronic Supporting Information

Natural Born Laser Dyes: Excited-State Intramolecular Proton Transfer (ESIPT) Emitters and Their Use in Random Lasing Studies

Julien Massue,[a]* Thibault Pariat,[a] Pauline M. Vérité,[b] Denis Jacquemin,[b]* Martyna Durko,[c] Tarek Chtouki,[c] Lech Sznitko,[c] Jaroslaw Mysliwiec[c]* and Gilles Ulrich[a]

S1. Synthetic schemes

S2. Experimental procedures

S3. ^{1}H and ^{13}C NMR traces

S4. HR-MS spectra

S5. Spectroscopic data

S6. Theoretical calculations

S7. Sample preparation for RL studies

S1. Synthetic schemes

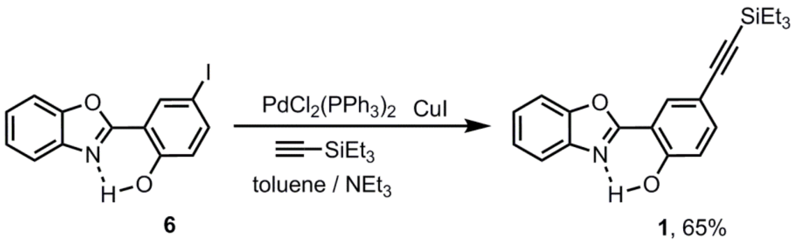

Scheme S1.1. Synthesis of HBO dye **1**.

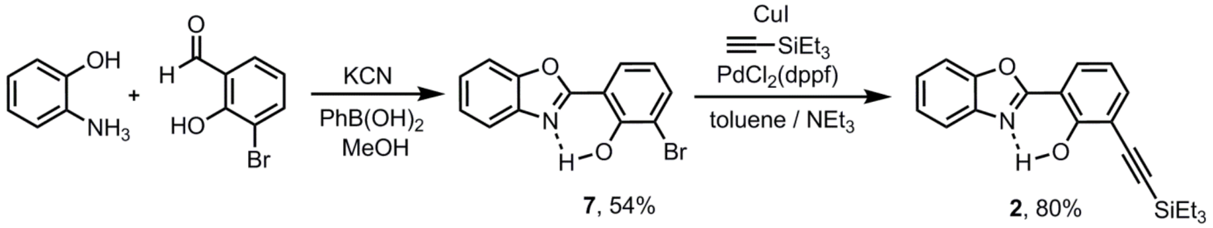

Scheme S1.2. Synthesis of HBO dye **2**.

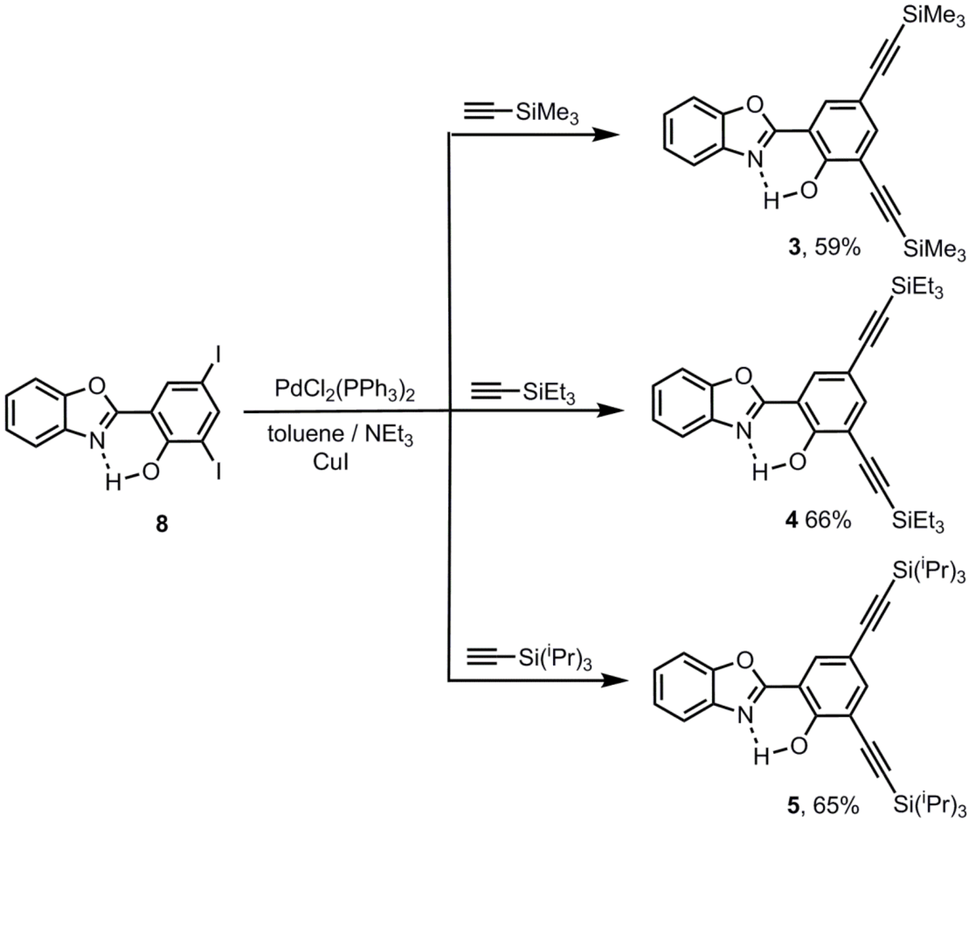

Scheme S1.3. Synthesis of HBO dyes **3**, **4** and **5**.

S2. Experimental procedures

Materials and methods

All reactions were performed under a dry atmosphere of argon. Chemicals were purchased from commercial sources and used without further purification. Reaction solvents were distilled according to common procedures. Thin layer chromatography (TLC) was performed on silica gel or aluminum oxide plates coated with fluorescent indicator. Chromatographic purifications were conducted using 40-63 μm silica gel. All mixtures of solvents are given in *v*/*v* ratio.

^{1}H NMR (400.1 MHz) and ^{13}C NMR (100.5 MHz) spectra were recorded on a Bruker Advance 400 MHz spectrometer, with perdeuterated solvents containing residual protonated solvent signals as internal references.

Absorption spectra were recorded using a dual-beam grating Schimadzu UV-3000 absorption spectrometer with a 1 cm quartz cell. The steady-state fluorescence emission and excitation spectra were obtained by using a Horiba S2 Jobin Yvon Fluoromax 4. All fluorescence spectra were corrected. Solvents for spectroscopy were spectroscopic grade and were used as received. All fluorescence spectra were corrected. The fluorescence quantum yield (Φ_{exp}) was calculated from Eq (1).

$$\Phi_{exp} = \Phi_{ref} \frac{I}{I_{ref}} \frac{OD_{ref}}{OD} \frac{\eta^2}{\eta^2_{ref}} \quad (eq\ 1)$$

where I denotes the integral of the corrected emission spectrum, OD is the optical density at the excitation wavelength, and η is the refractive index of the medium. The quantum yield was determined in solution by using quinine sulfate as a reference (λ_{exc} = 366 nm, Φ = 0.55 in 1N H_2SO_4), for dyes emitting below 480 nm, Rhodamine 6G as a reference (λ_{exc} = 488 nm, Φ =

0.88 in ethanol), for dyes emitting between 480 and 570 nm or cresyl violet (λ_{exc} = 546 nm, Φ = 0.55 in ethanol) as a reference for dyes emitting above 570 nm.

Luminescence lifetimes were measured on an Edinburgh Instruments spectrofluorimeter equipped with a R928 photomultiplier and a PicoQuant PDL 800-D pulsed diode connected to a GwInstect GFG- 8015G delay generator. No filter was used for the excitation. Emission wavelengths were selected by a monochromator. Lifetimes were deconvoluted with FS-900 software using a light-scattering solution (LUDOX) for instrument response. The excitation source was a laser diode (λ = 320 nm).

HBO dyes **3**, **6** and **8** were synthesized according to reported procedures.[1,2]

Synthetic procedures

Synthesis of HBO **1**

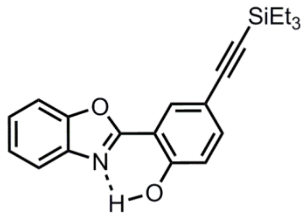

1

To a degassed solution of HBO **6** (1 eqt.) and $Pd(PPh_3)_2Cl_2$ (5 % mol.) in toluene and triethylamine (3/1, *v/v*), was added (triethylsilyl)acetylene (3 eqts.) and CuI (10 % mol.). The resulting mixture was stirred overnight at 90°C. After cooling down, it was extracted with CH_2Cl_2, washed with water three times. The organic layers were dried over $MgSO_4$ and evaporated *in vacuo*. The product was purified by column chromatography on SiO_2 (CH_2Cl_2/ Pet. Ether. 1:9) to afford clean HBO **1** as a white powder. 65%. ^{1}H NMR (400MHz, $CDCl_3$) δ (ppm): 11.66 (s, 1H, OH), 8.16 (d, 1H, CH, ^4J = 2Hz), 7.70-7.74 (m, 1H, CH), 7.59-7.63 (m, 1H, CH), 7.54 (dd, 1H, CH, ^3J = 8.8Hz, ^4J = 2Hz), 7.36-7.41 (m, 2H, CH), 7.05 (d, 1H, CH, ^3J = 8.4Hz), 1.09 (t, 9H, CH_3, ^3J = 7.6Hz), 0.71 (q, 6H, CH_2, ^3J = 8.4Hz). ^{13}C NMR (100.5Hz, $CDCl_3$) δ (ppm): 161.1, 157.7, 148.1, 138.8, 136.1, 129.9, 124.7, 124.2, 118.3, 116.6, 113.8, 109.7, 109.5, 104.3, 89.5, 6.5, 3.5.ESI-HRMS: calcd for $C_{21}H_{24}NO_2Si$: 350.1571 (M+H), found 350.1570 (M+H).

Synthesis of HBO **7**

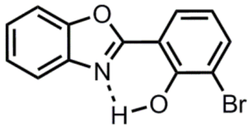

7

A mixture of 2-hydroxy-3-bromobenzaldehyde and 2-aminophenol (1 eqt) were refluxed in EtOH for 3 hours. An orange precipitate rapidly appeared that was filtered and further washed with EtOH before being redissolved in distilled CH_2Cl_2. 2,6-dichloro-3,5-dicyano-1,4-benzoquinone (DDQ) (1.2 eqt) was then added by portions and the resulting mixture stirred overnight at room temperature. The solvents were removed *in vacuo* and the crude mixture purified by column chromatography on SiO_2 (CH_2Cl_2/Pet. Ether. 1:9) to avoid HBO **7** as a white powder. 54%. ^{1}H NMR (400MHz, $CDCl_3$) δ (ppm): 12.27 (s, 1H, OH), 8.01 (dd, 1H, CH, ^3J = 7.6Hz, ^4J = 1.6Hz), 7.73-7.78 (m, 1H, CH), 7.70 (dd, 1H, CH, ^3J = 7.6Hz, ^4J = 1.6Hz), 7.60-7.65 (m, 1H, CH), 7.39-7.44 (m, 2H, CH), 6.93 (t, 1H, CH, ^3J = 8Hz). ^{13}C NMR (100.5Hz, $CDCl_3$) δ (ppm): 162.3, 155.6, 149.5, 139.8, 137.0, 126.5, 126.1, 125.5, 120.6, 119.7, 111.9, 111.5, 111.0. ESI-HRMS: calcd for $C_{13}H_9BrNO_2$: 291.9792 (M+H), found 291.9775 (M+H).

Synthesis of HBO **2**

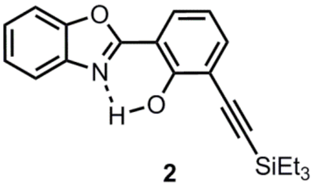

2

To a degassed solution of HBO **7** (1 eqt.) and $Pd(PPh_3)_2Cl_2$ (5 % mol.) in toluene and triethylamine (3/1, *v/v*), was added (triethylsilyl)acetylene (3 eqts.) and CuI (10 % mol.). The resulting mixture was stirred overnight at 90°C. After cooling down, it was extracted with CH_2Cl_2, washed with water three times. The organic layers were dried over $MgSO_4$ and evaporated *in vacuo*. The product was purified by column chromatography on SiO_2 (CH_2Cl_2/ Pet. Ether. 1:9) to afford clean HBO **2** as a white powder. 80%. ^{1}H NMR (400MHz, $CDCl_3$) δ (ppm): 12.07 (s, 1H, OH), 8.00 (dd, 1H, CH, ^3J = 8Hz, ^4J = 1.6Hz), 7.70-7.75 (m, 1H, CH), 7.59-7.63 (m, 2H, CH), 7.37-7.42 (m, 2H, CH), 6.95 (t, 1H, CH, ^3J = 7.6Hz), 1.10 (t, 9H, CH_3, ^3J = 7.6 Hz), 0.73 (q, 6H, CH_2, ^3J = 6.8 Hz). ^{13}C NMR (100.5Hz, $CDCl_3$) δ (ppm): 162.5, 159.8, 149.2, 139.8, 137.5, 127.3, 125.6, 125.2, 119.4, 119.1, 112.8, 110.8, 110.7, 101.3, 97.3, 7.6, 4.5. ESI-HRMS: calcd for $C_{21}H_{24}NO_2Si$: 350.1571 (M+H), found 350.1576 (M+H).

Synthesis of HBO **4**

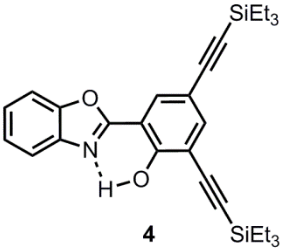

4

To a degassed solution of HBO **8** (1 eqt.) and $Pd(PPh_3)_2Cl_2$ (5 % mol.) in toluene and triethylamine (3/1, *v/v*), was added (triethylsilyl)acetylene (6 eqts.) and CuI (10 % mol.). The resulting mixture was stirred overnight at 90°C. After cooling down, it was extracted with CH_2Cl_2, washed with water three times. The organic layers were dried over $MgSO_4$ and evaporated *in vacuo*. The product was purified by column chromatography on SiO_2 (CH_2Cl_2/ Pet. Ether. 1:9) to afford clean HBO **4** as a white powder. 66%. ^{1}H NMR (400MHz, $CDCl_3$) δ (ppm): 12.24 (s, 1H, OH), 8.10 (d, 1H, CH, ^4J = 2Hz), 7.71-7.74 (m, 1H, CH), 7.70 (d, 1H, CH, ^4J = 2Hz), 7.59-7.64 (m, 1H, CH), 7.37-7.43 (m, 2H, CH), 1.10 (t, 9H, CH_3, ^3J = 7.6Hz), 1.08 (t, 9H, CH_3, ^3J = 7.6Hz), 0.72 (q, 6H, CH_2, ^3J = 8.4Hz), 0.70 (q, 6H, CH_2, ^3J = 12Hz). ^{13}C NMR (100.5Hz, $CDCl_3$) δ (ppm): 161.7, 159.7, 149.2, 140.5, 139.6, 130.7, 125.9, 125.3, 119.5, 114.6, 113.2, 110.8, 104.5, 100.3, 97.9, 91.1, 7.6, 4.5. ESI-HRMS: calcd for $C_{29}H_{38}NO_2Si_2$: 488.2436 (M+H), found 488.2438 (M+H).

Synthesis of HBO **5**

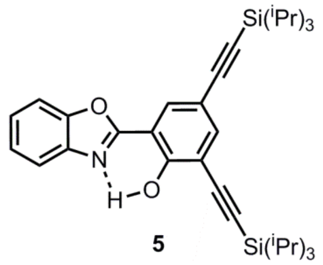

5

To a degassed solution of HBO **8** (1 eqt.) and $Pd(PPh_3)_2Cl_2$ (5 % mol.) in toluene and triethylamine (3/1, *v/v*), was added (triisopropylsilyl)acetylene (6 eqts.) and CuI (10 % mol.). The resulting mixture was stirred overnight at 90°C. After cooling down, it was extracted with CH_2Cl_2, washed with water three times. The organic layers were dried over $MgSO_4$ and evaporated *in vacuo*. The product was purified by column chromatography on SiO_2 (CH_2Cl_2/ Pet. Ether. 1:9) to afford clean HBO **5** as a white powder. 65%. ^{1}H NMR (400MHz, $CDCl_3$) δ (ppm): 12.19 (s, 1H, OH), 8.09 (d, 1H, CH, ^4J = 2Hz), 7.71-7.75 (m, 1H, CH), 7.67 (d, 1H, CH, ^4J = 2Hz), 7.62-7.66 (m, 1H, CH), 7.38-7.43 (m, 2H, CH), 1.17 (d, 42H, CH + CH_3, ^3J = 9.2Hz). ^{13}C NMR (100.5Hz, $CDCl_3$) δ (ppm): 161.8, 159.9, 149.2, 140.3, 139.7, 130.6, 125.9, 125.3, 119.4, 114.7, 113.4, 110.8, 110.8, 105.3, 101.0, 97.0, 18.7, 11.4. ESI-HRMS: calcd for $C_{35}H_{50}NO_2Si_2$: 572.3375 (M+H), found 572.3429 (M

S3 ^{1}H and ^{13}C NMR traces

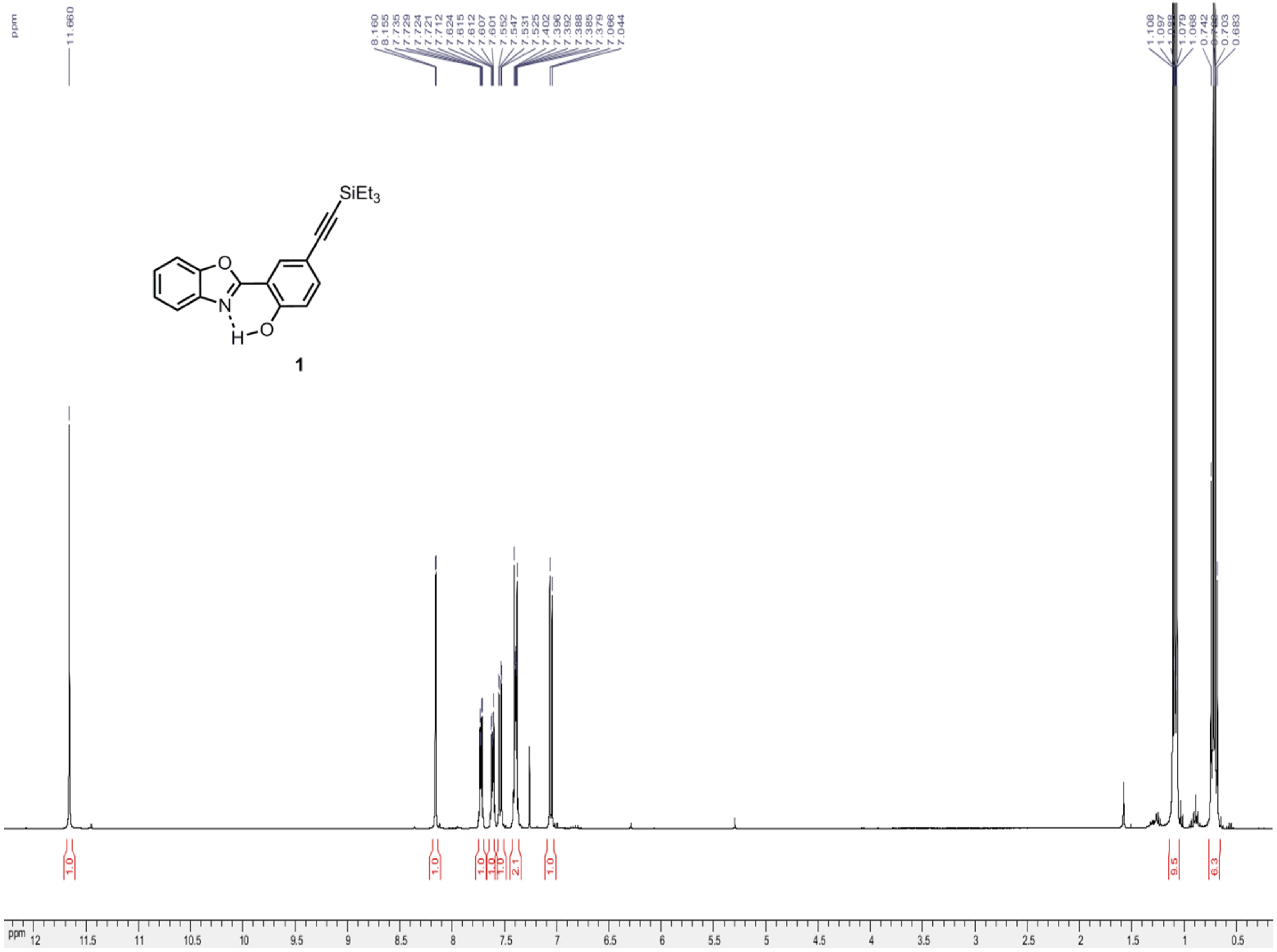

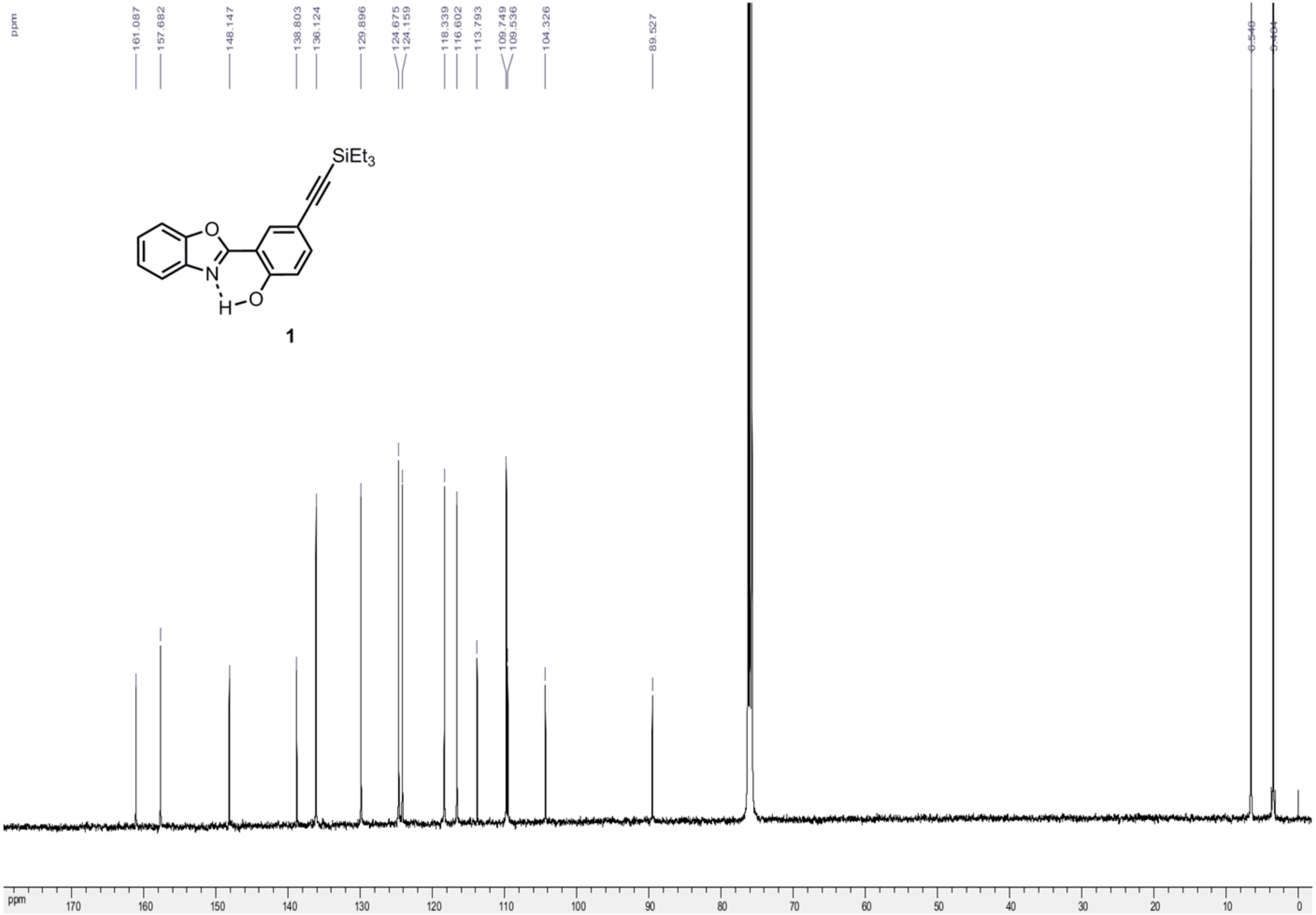

ppm
161.087
157.682
148.147
138.803
136.124
129.896
124.675
124.159
118.339
116.602
113.793
109.749
109.536
104.326
89.527
6.540
3.404
SiEt3
O
N
H
O
1
ppm
170
160
150
140
130
120
110
100
90
80
70
60
50
40
30
20
10
0

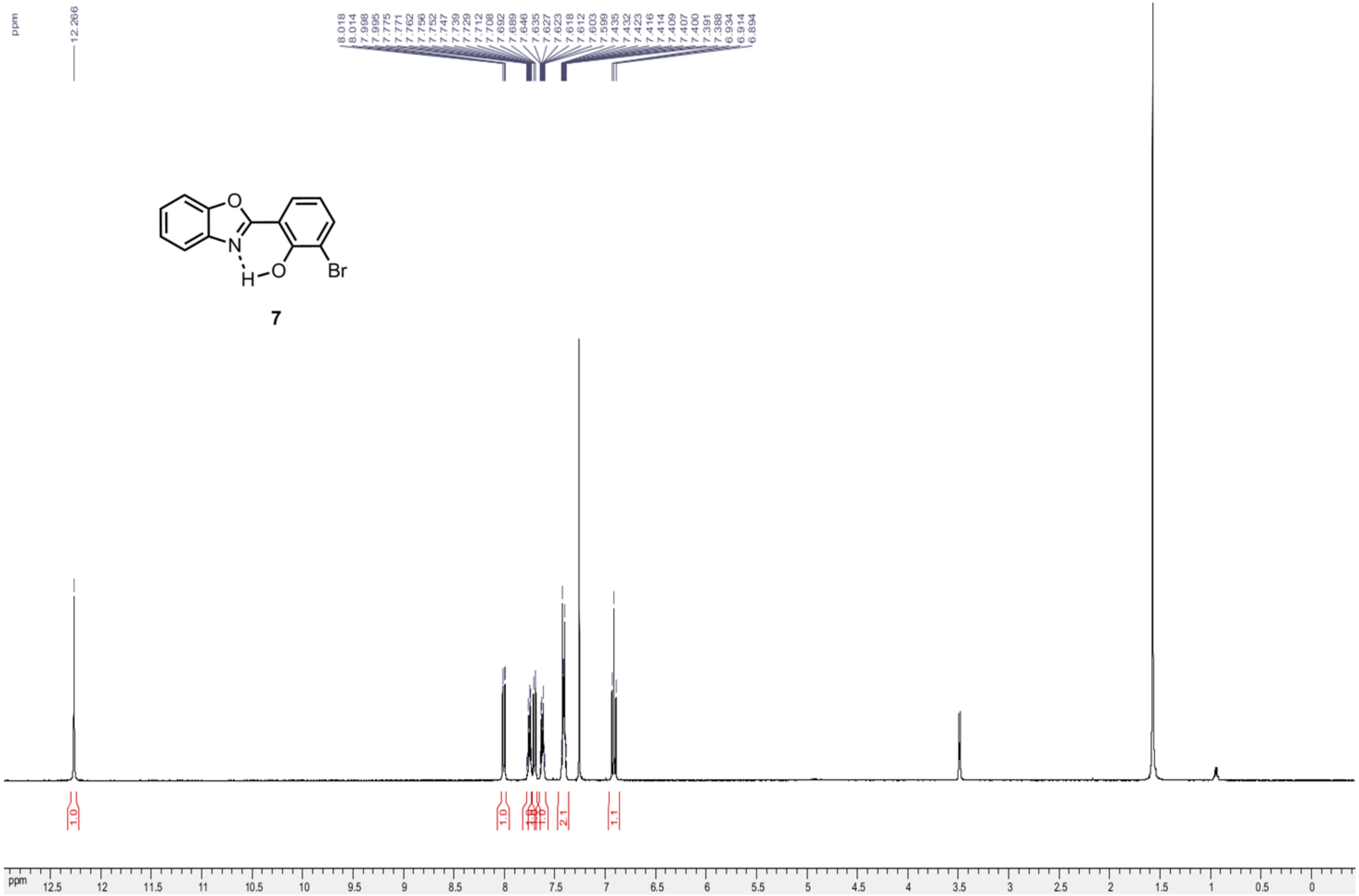
ppm
12.266
8.018 8.014 7.998 7.995 7.775 7.771 7.762 7.756 7.752 7.747 7.739 7.729 7.712 7.708 7.692 7.689 7.646 7.635 7.627 7.623 7.618 7.612 7.603 7.599 7.435 7.432 7.423 7.416 7.414 7.409 7.407 7.400 7.391 7.383 6.934 6.914 6.894
O
N
H
O
Br
7
1.0
1.0
2.1
1.1
ppm 12.5 12 11.5 11 10.5 10 9.5 9 8.5 8 7.5 7 6.5 6 5.5 5 4.5 4 3.5 3 2.5 2 1.5 1 0.5 0

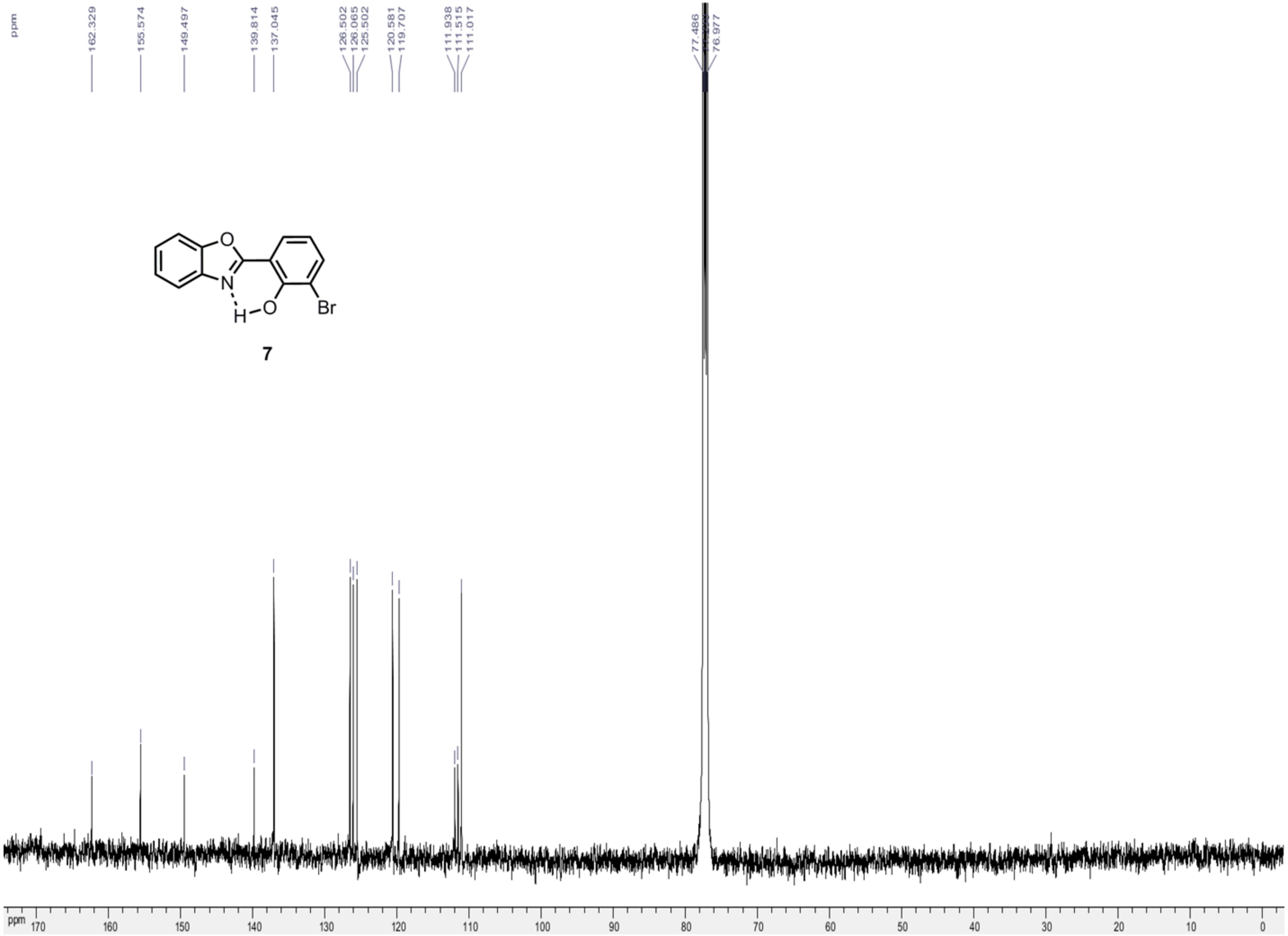

ppm
162.329
155.574
149.497
139.814
137.045
126.502
126.065
125.502
120.581
119.707
111.938
111.515
111.017
77.486
76.977
ppm 170 160 150 140 130 120 110 100 90 80 70 60 50 40 30 20 10 0

7

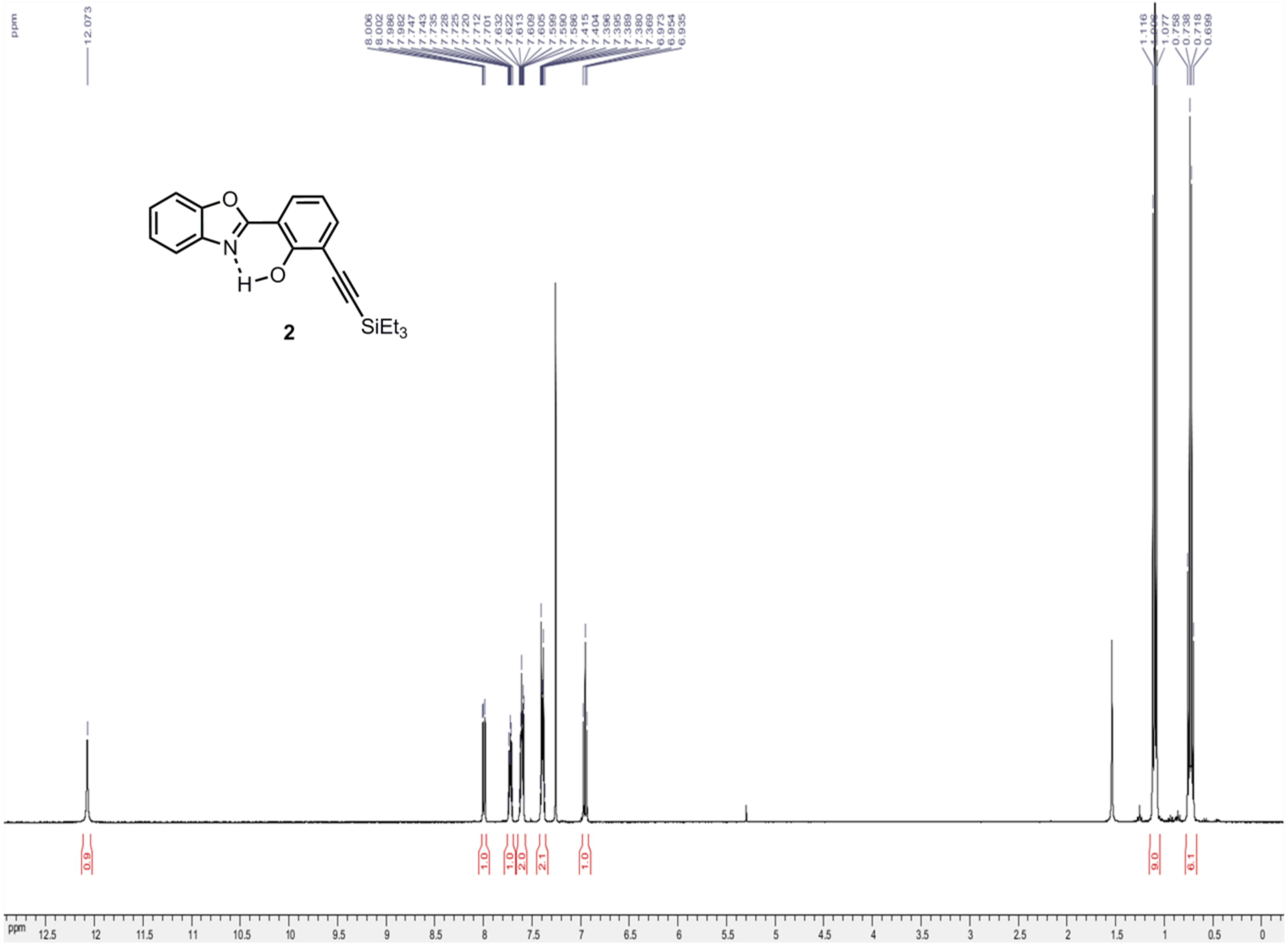

ppm
12.073
8.006
8.002
7.986
7.982
7.747
7.743
7.735
7.728
7.725
7.720
7.712
7.701
7.632
7.622
7.613
7.609
7.605
7.599
7.590
7.586
7.415
7.404
7.396
7.395
7.389
7.380
7.369
6.973
6.954
6.935
1.116
1.077
0.758
0.738
0.718
0.699
O
N
H
O
SiEt3
2
0.9
1.0
1.0
2.0
2.1
1.0
9.0
6.1
ppm 12.5 12 11.5 11 10.5 10 9.5 9 8.5 8 7.5 7 6.5 6 5.5 5 4.5 4 3.5 3 2.5 2 1.5 1 0.5 0

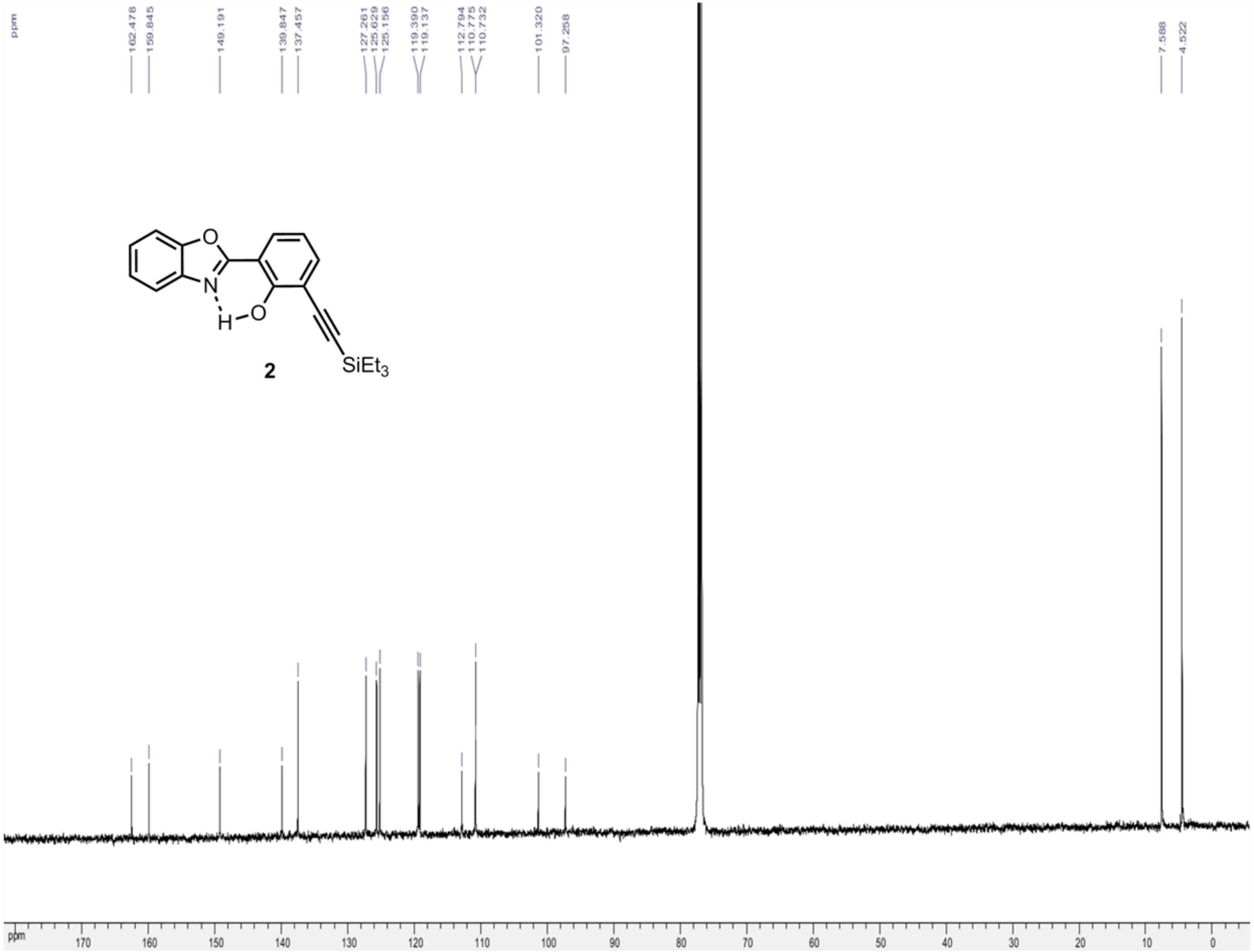
ppm
162.478
159.845
149.191
139.847
137.457
127.261
125.629
125.156
119.390
119.137
112.794
110.775
110.732
101.320
97.258
7.588
4.522
O
N
H
O
SiEt3
2
ppm
170
160
150
140
130
120
110
100
90
80
70
60
50
40
30
20
10
0

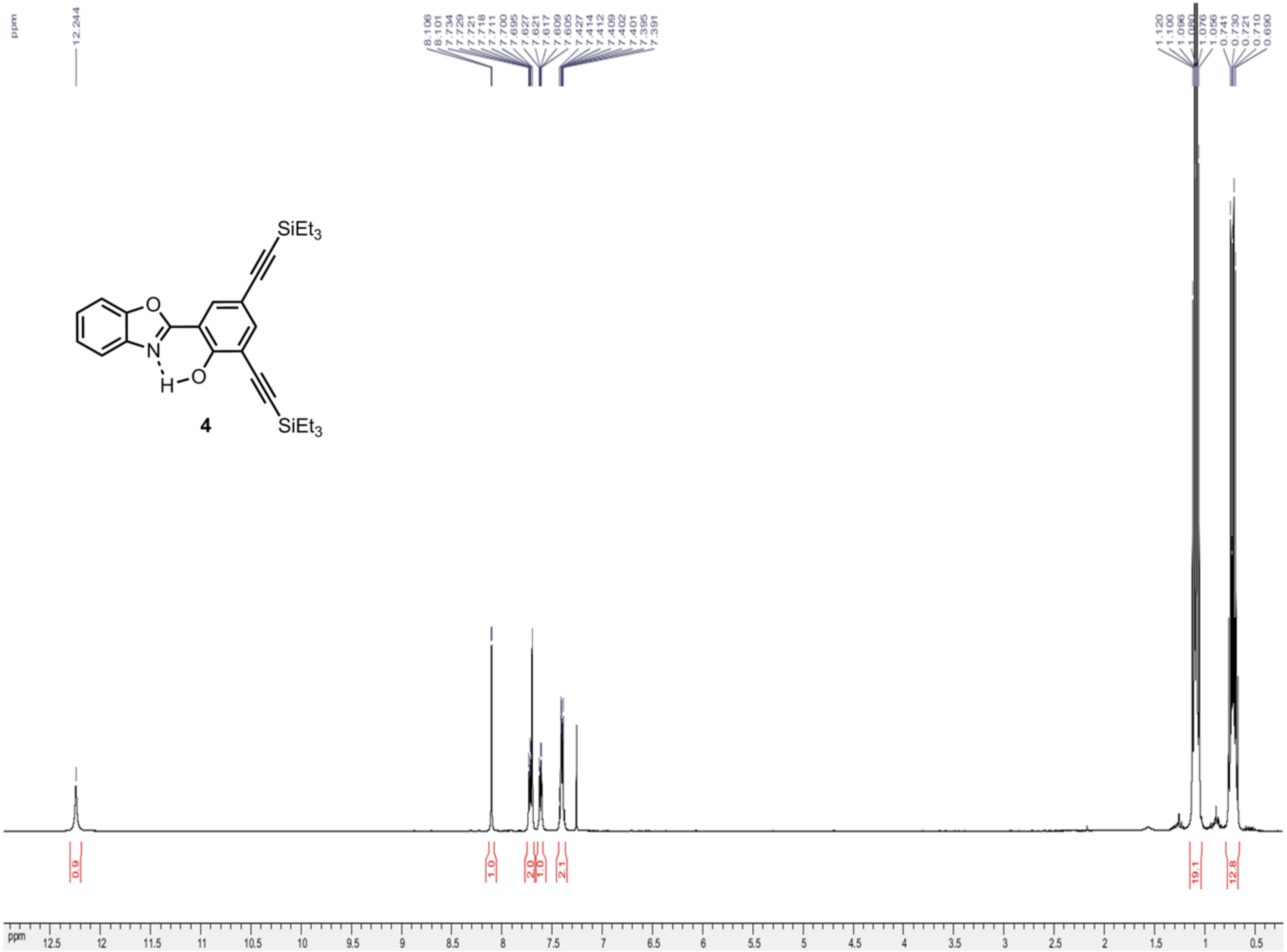

ppm
12.244
8.106
8.101
7.734
7.729
7.721
7.718
7.711
7.700
7.695
7.627
7.621
7.617
7.609
7.605
7.427
7.414
7.412
7.409
7.402
7.401
7.395
7.391
1.120
1.100
1.096
1.080
1.076
1.056
0.741
0.730
0.721
0.710
0.690
SiEt3
O
N
H
O
SiEt3
4
0.9
1.0
2.0
1.0
2.1
19.1
12.8
ppm
12.5
12
11.5
11
10.5
10
9.5
9
8.5
8
7.5
7
6.5
6
5.5
5
4.5
4
3.5
3
2.5
2
1.5
1
0.5

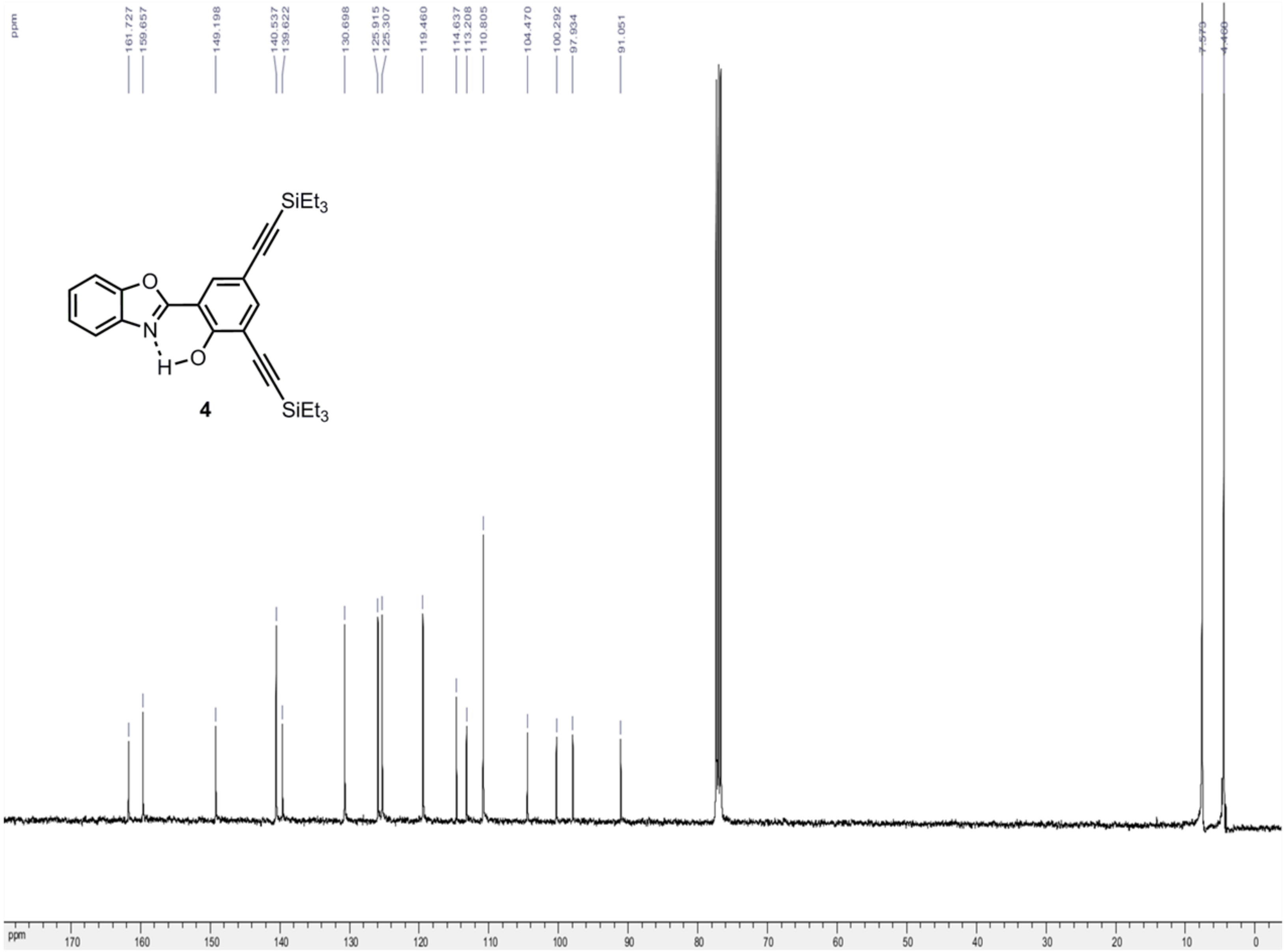
ppm
161.727
159.657
149.198
140.537
139.622
130.698
125.915
125.307
119.460
114.637
113.208
110.805
104.470
100.292
97.934
91.051
7.573
4.460
SiEt3
O
N
H
O
SiEt3
ppm 170 160 150 140 130 120 110 100 90 80 70 60 50 40 30 20 10 0

4

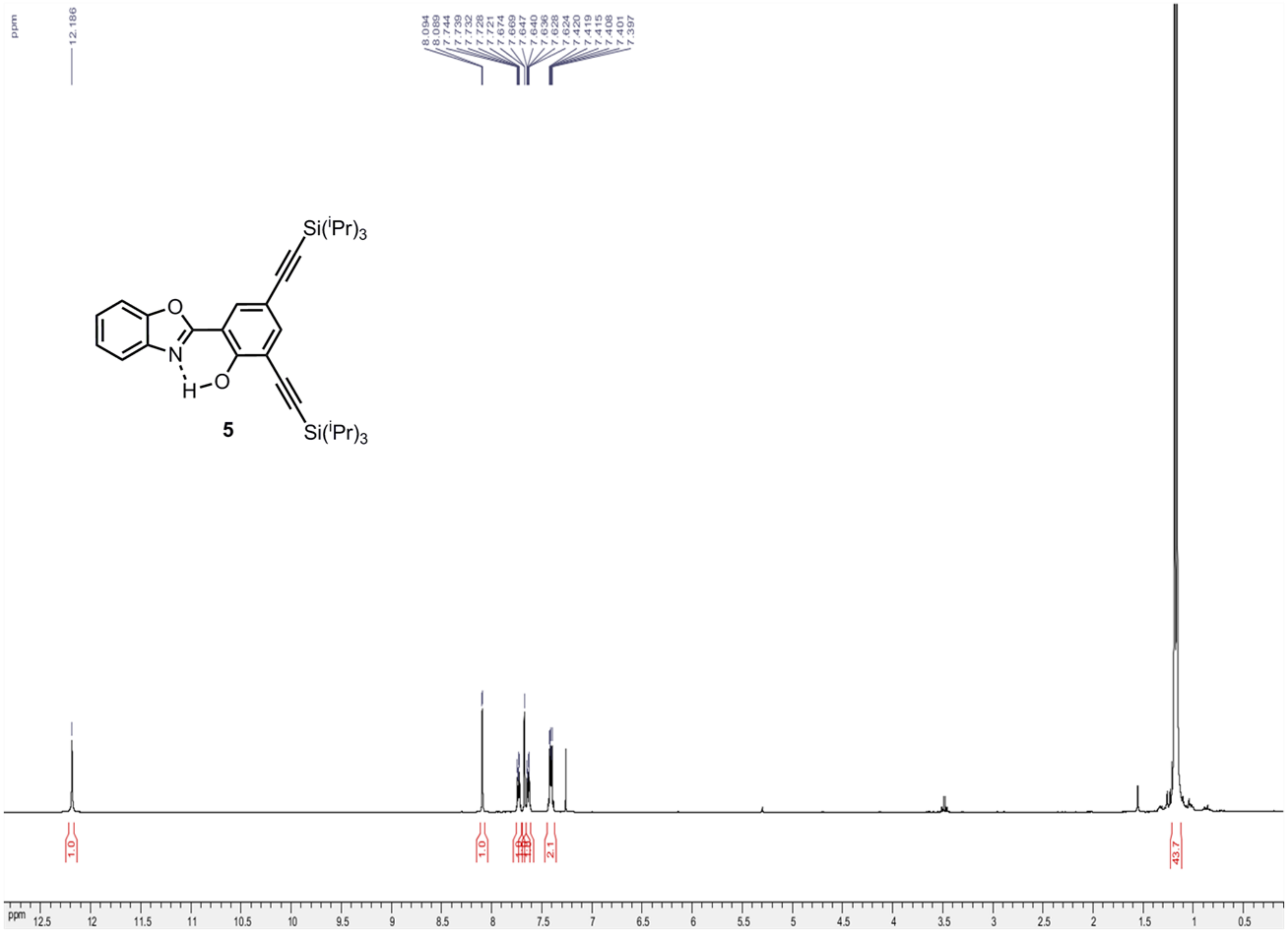

ppm
12.186
8.094
8.089
7.744
7.739
7.732
7.728
7.721
7.674
7.669
7.647
7.640
7.636
7.628
7.624
7.420
7.419
7.415
7.408
7.401
7.397
Si(iPr)3
O
N
H
O
5
Si(iPr)3
1.0
1.0
2.1
43.7
ppm 12.5 12 11.5 11 10.5 10 9.5 9 8.5 8 7.5 7 6.5 6 5.5 5 4.5 4 3.5 3 2.5 2 1.5 1 0.5

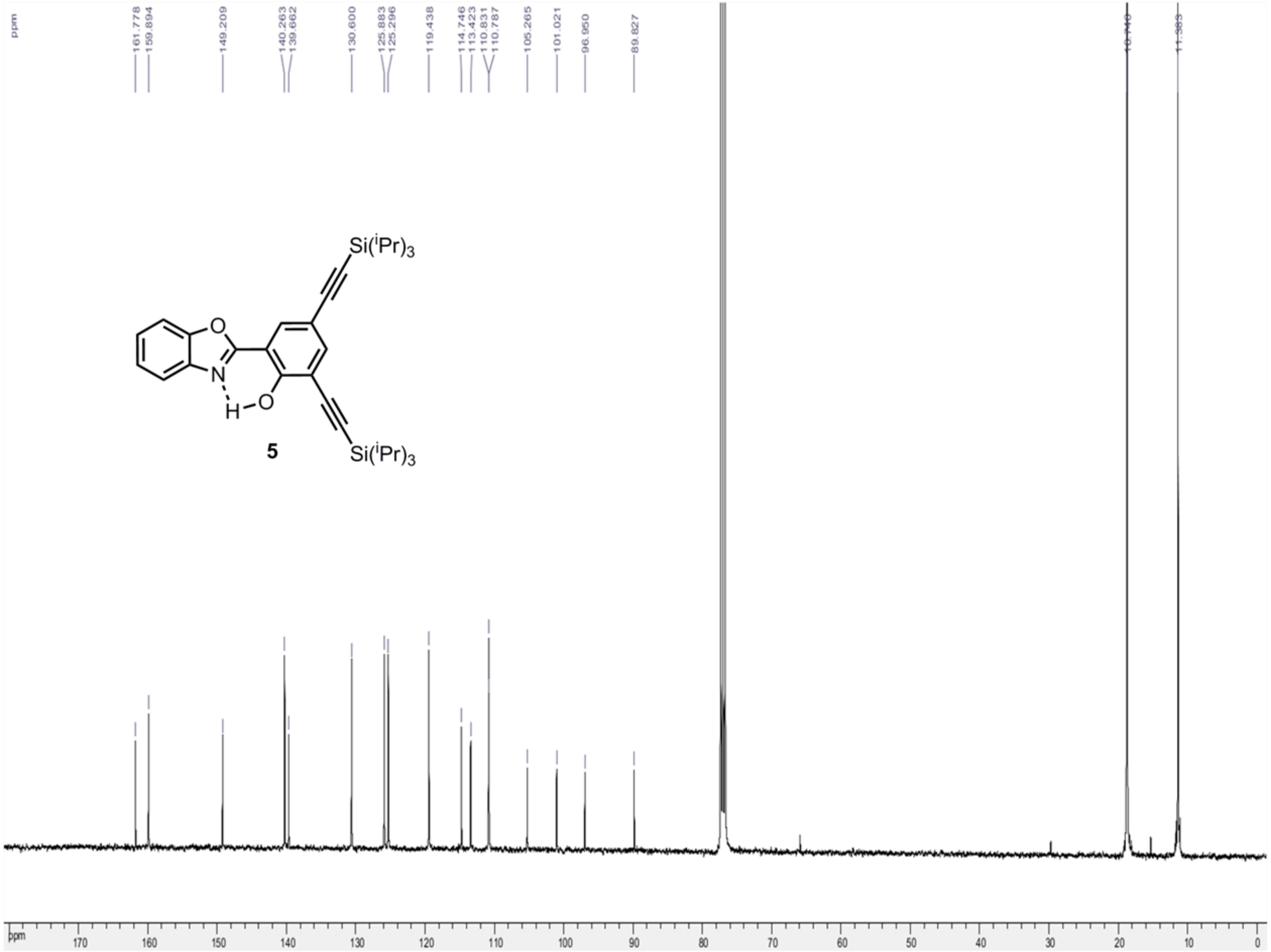
ppm
161.778
159.894
149.209
140.263
139.662
130.600
125.883
125.296
119.438
114.746
113.423
110.831
110.787
105.265
101.021
96.950
89.827
11.383
Si(iPr)3
O
N
H
O
5
Si(iPr)3
ppm
170
160
150
140
130
120
110
100
90
80
70
60
50
40
30
20
10
0

S4 HRMS spectra

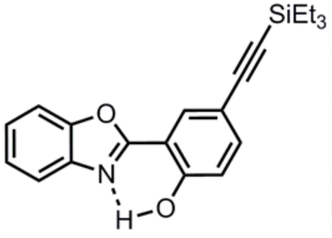

1

Mass Spectrum HR Report

a\SM 2017\F00513SK.d
pos_Standard.m

Acquisition Date	5/30/2017 1:50:26 PM	
Operator	BDAL@DE	
Instrument	micrOTOF II	8213750.1045 1

Acquisition Parameter

				Set Corrector Fill	59.0 V
Source Type	ESI	Ion Polarity	Positive	n/a	n/a
n/a	n/a	n/a	n/a	n/a	n/a
Scan Begin	50 m/z	n/a	n/a	Set Reflector	1800.0 V
Scan End	3000 m/z	n/a	n/a	Set Flight Tube	8600.0 V
		n/a	n/a	Set Detector TOF	1953.3 V

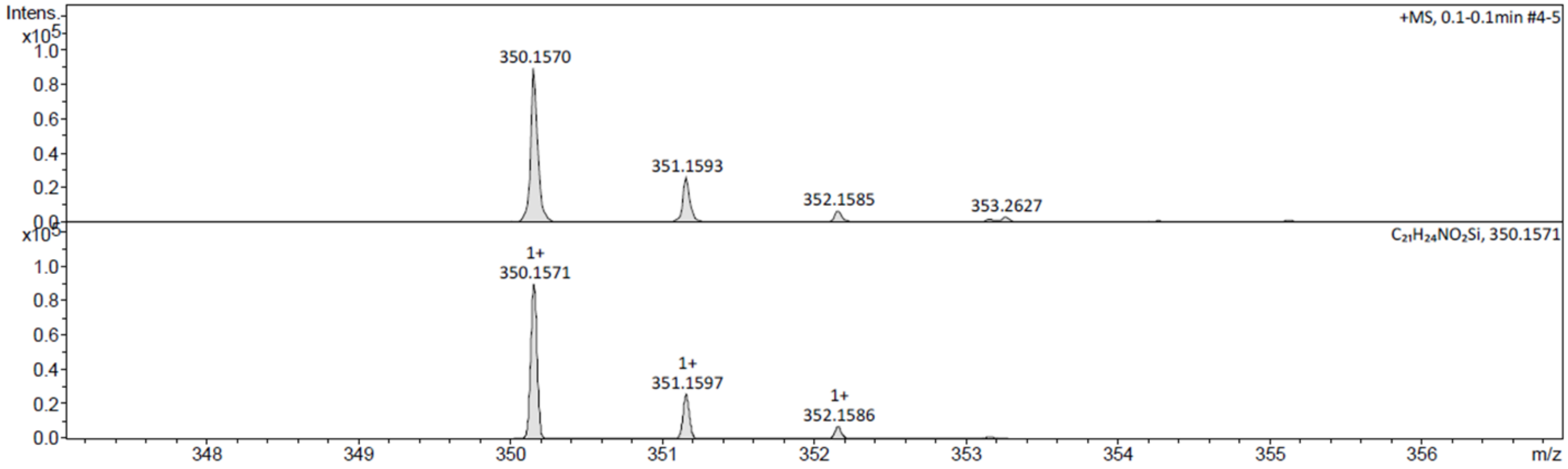

Mass Spectrum HR Report

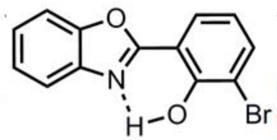

7

:\Data\SM 2017\F00277SK.d	Acquisition Date	4/28/2017 9:55:01 AM		
une_pos_Standard.m	Operator	BDAL@DE		
ICDE062	Instrument	micrOTOF II	8213750.1045<br>1	

Acquisition Parameter

				Set Corrector Fill	59.0 V
Source Type	ESI	Ion Polarity	Positive	n/a	n/a
n/a	n/a	n/a	n/a	n/a	n/a
Scan Begin	50 m/z	n/a	n/a	Set Reflector	1800.0 V
Scan End	3000 m/z	n/a	n/a	Set Flight Tube	8600.0 V
		n/a	n/a	Set Detector TOF	1953.3 V

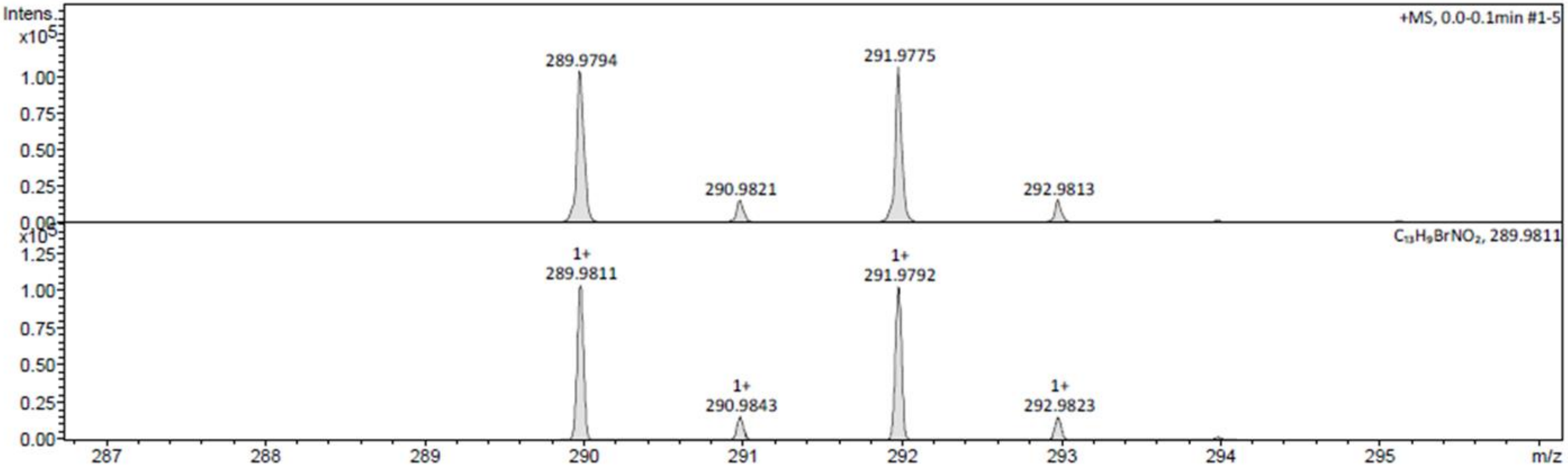

Meas. m/z	#	Ion Formula	m/z	err [ppm]	Mean err [ppm]	rdb	N-Rule	e⁻ Conf	mSigma	Std I	Std Mean m/z	Std I VarNorm	Std m/z Diff	Std Comb Dev
289.979405	1	C13H9BrNO2	289.981117	5.9	5.7	9.5	ok	even	13.2	12.3	n.a.	n.a.	n.a.	n.a.

Mass Spectrum HR Report

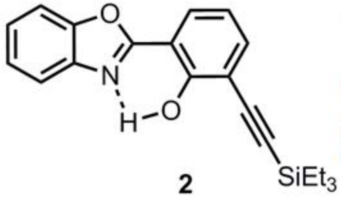

2

\SMasse\2017\Juin 2017\F00596SK.d
os_Standard.m
77

Acquisition Date	6/7/2017 10:56:08 AM
Operator	BDAL@DE
Instrument	micrOTOF II 8213750.1045 1

Acquisition Parameter

Source Type	ESI	Ion Polarity	Positive	Set Corrector Fill	59.0 V
n/a	n/a	n/a	n/a	n/a	n/a
Scan Begin	50 m/z	n/a	n/a	n/a	n/a
Scan End	3000 m/z	n/a	n/a	Set Reflector	1800.0 V
		n/a	n/a	Set Flight Tube	8600.0 V
		n/a	n/a	Set Detector TOF	1953.3 V

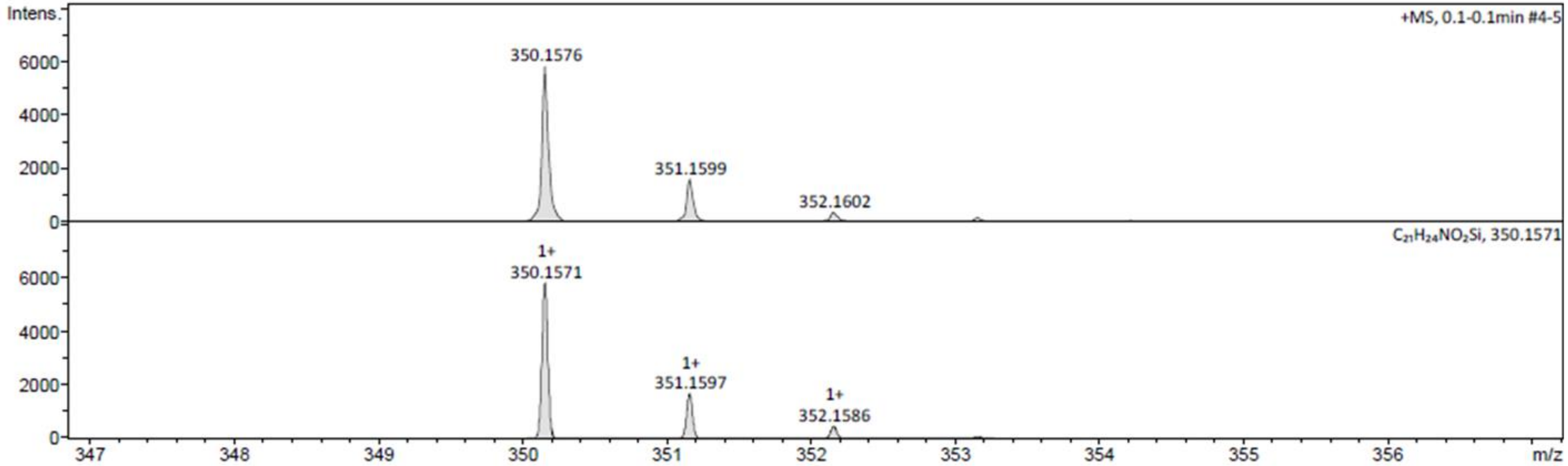

Meas. m/z	#	Ion Formula	m/z	err [ppm]	Mean err [ppm]	rdb	N-Rule	e⁻ Conf	mSigma	Std I	Std Mean m/z	Std I VarNorm	Std m/z Diff	Std Comb Dev
350.157578	1	C21H24NO2Si	350.157082	-1.4	1411.2	11.5	ok	even	13.1	28.9	n.a.	n.a.	n.a.	n.a.
	2	C21H22NO2Si	348.141432	-2911.2	-170.5	12.5	ok	even	914.0	853.9	n.a.	n.a.	n.a.	n.a.

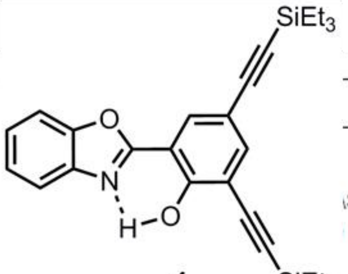

4

Mass Spectrum Molecular Formula Report

Acquisition Date	3/10/2017 9:47:50 AM
Operator	Administrator
Instrument	micrOTOF 66

\Service masse 2017\O41805SK.d
pos.m

Acquisition Parameter

				Set Corrector Fill	57 V
Source Type	ESI	Ion Polarity	Positive	Set Pulsar Pull	811 V
Scan Range	n/a	Capillary Exit	150.0 V	Set Pulsar Push	811 V
Scan Begin	50 m/z	Hexapole RF	220.0 V	Set Reflector	1700 V
Scan End	3000 m/z	Skimmer 1	50.0 V	Set Flight Tube	8600 V
		Hexapole 1	24.3 V	Set Detector TOF	1950 V

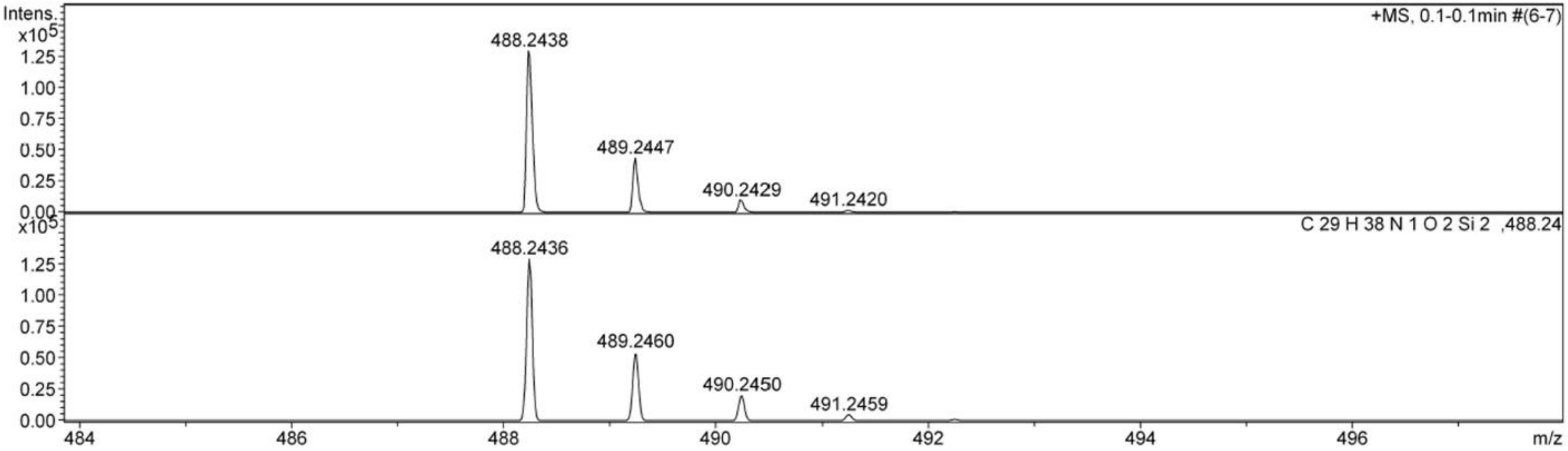

Sum Formula	Sigma	m/z	Err [ppm]	Mean Err [ppm]	rdb	N Rule	e⁻
C 29 H 38 N 1 O 2 Si 2	0.06	488.2436	-0.40	1.11	13.50	ok	even
C 29 H 37 N 1 O 2 Si 2	0.58	487.2357	-11.46	-12.08	14.00	-	odd

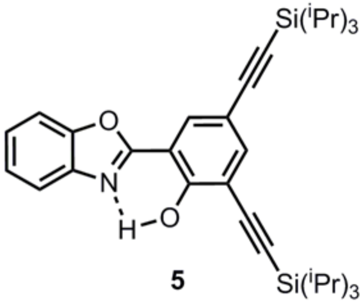

5

Mass Spectrum Molecular Formula Report

Service masse 2017\O41927SK.d		Acquisition Date	3/21/2017 3:03:40 PM
pos.m		Operator	Administrator
		Instrument	micrOTOF 66

Acquisition Parameter

				Set Corrector Fill	57 V
Source Type	ESI	Ion Polarity	Positive	Set Pulsar Pull	811 V
Scan Range	n/a	Capillary Exit	150.0 V	Set Pulsar Push	811 V
Scan Begin	50 m/z	Hexapole RF	220.0 V	Set Reflector	1700 V
Scan End	3000 m/z	Skimmer 1	50.0 V	Set Flight Tube	8600 V
		Hexapole 1	24.3 V	Set Detector TOF	1950 V

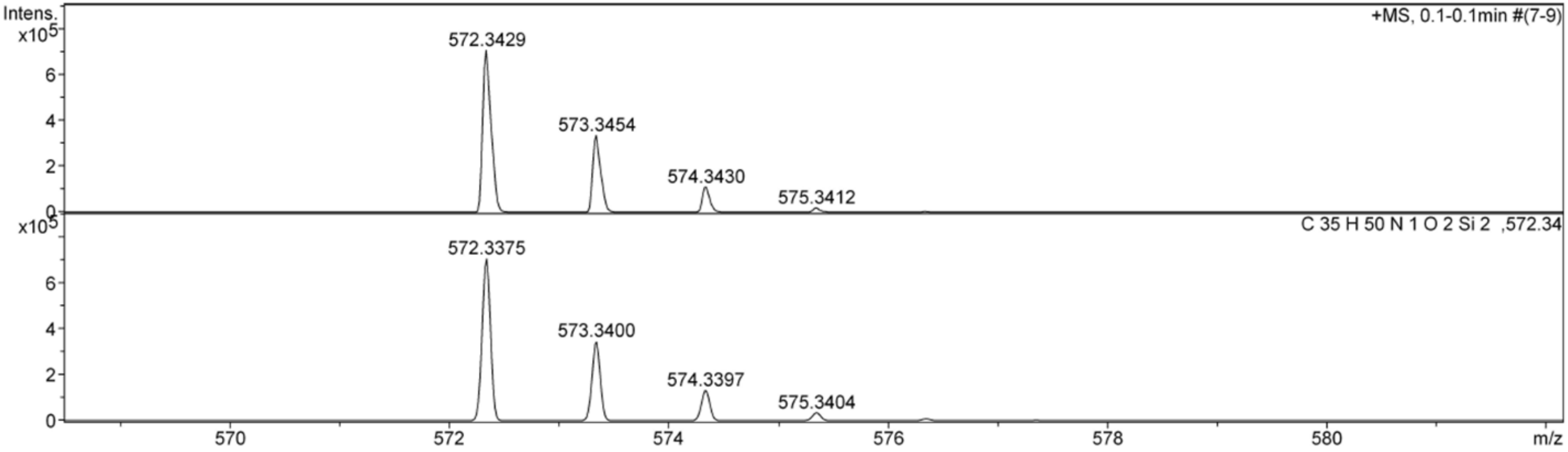

Sum Formula	Sigma	m/z	Err [ppm]	Mean Err [ppm]	rdb	N Rule	e⁻
C 35 H 50 N 1 O 2 Si 2	0.02	572.3375	-9.58	-8.75	13.50	ok	even

S5 Spectroscopic data

S5.1 Absorption, emission and excitation spectra of HBO dyes 1-5 in solution at 25°C

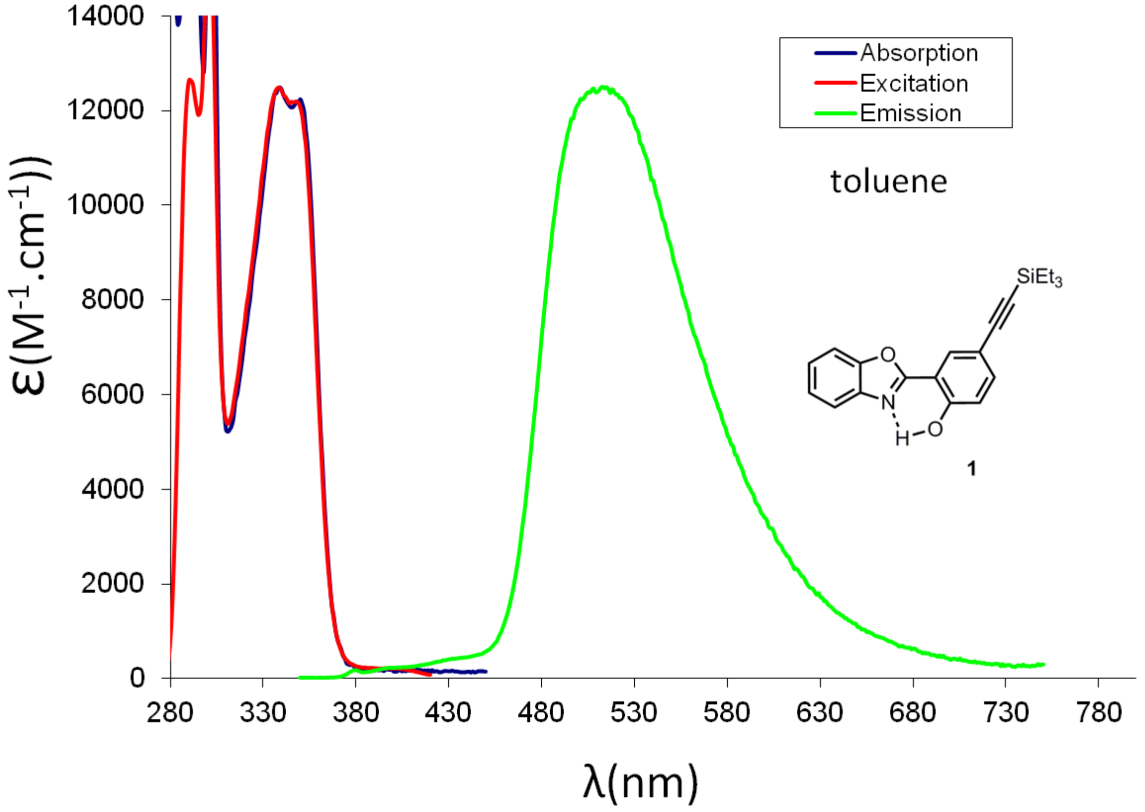

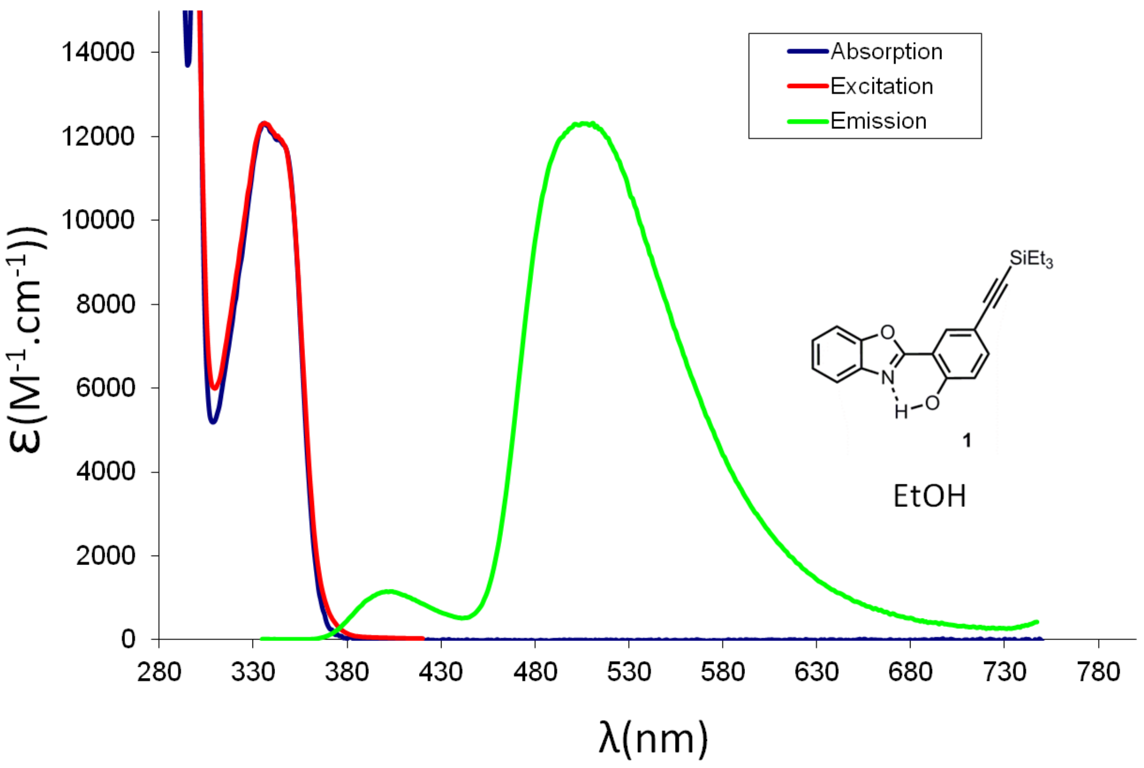

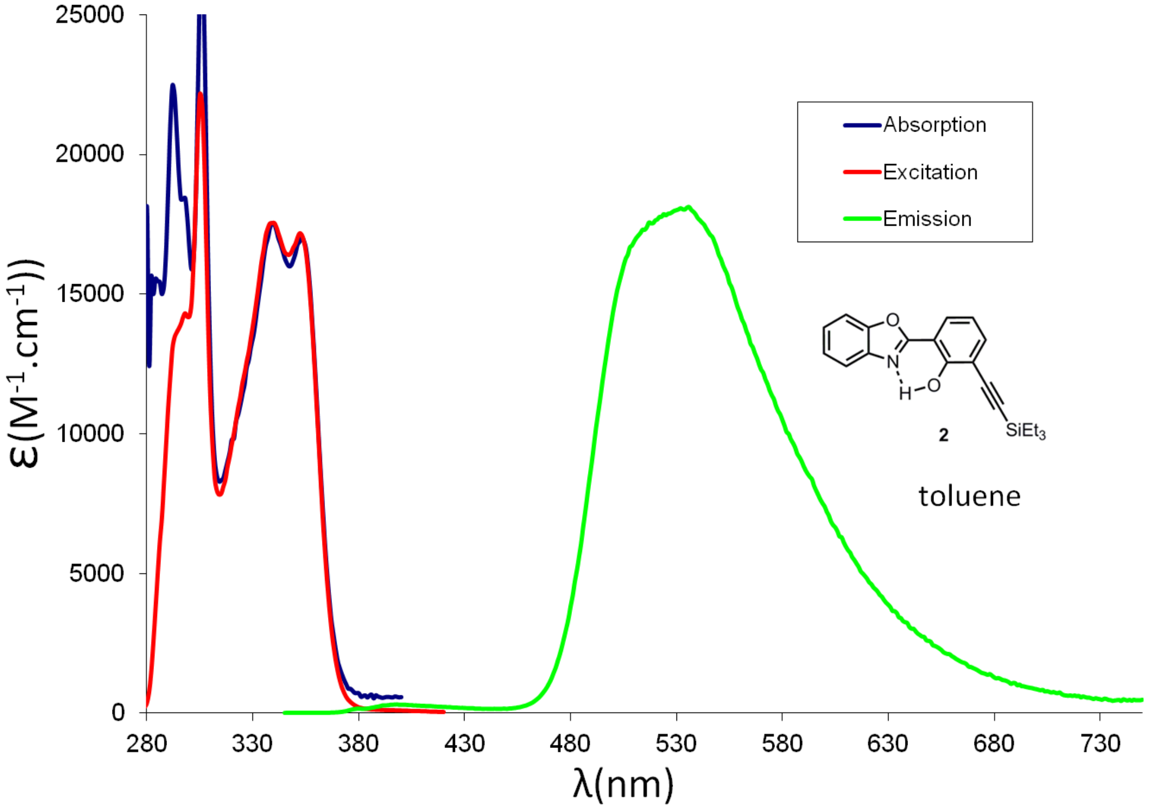
25000
20000
15000
10000
5000
0
ε(M-1.cm-1))
280
330
380
430
480
530
580
630
680
730
λ(nm)
Absorption
Excitation
Emission
O
N
H
O
2
SiEt3
toluene

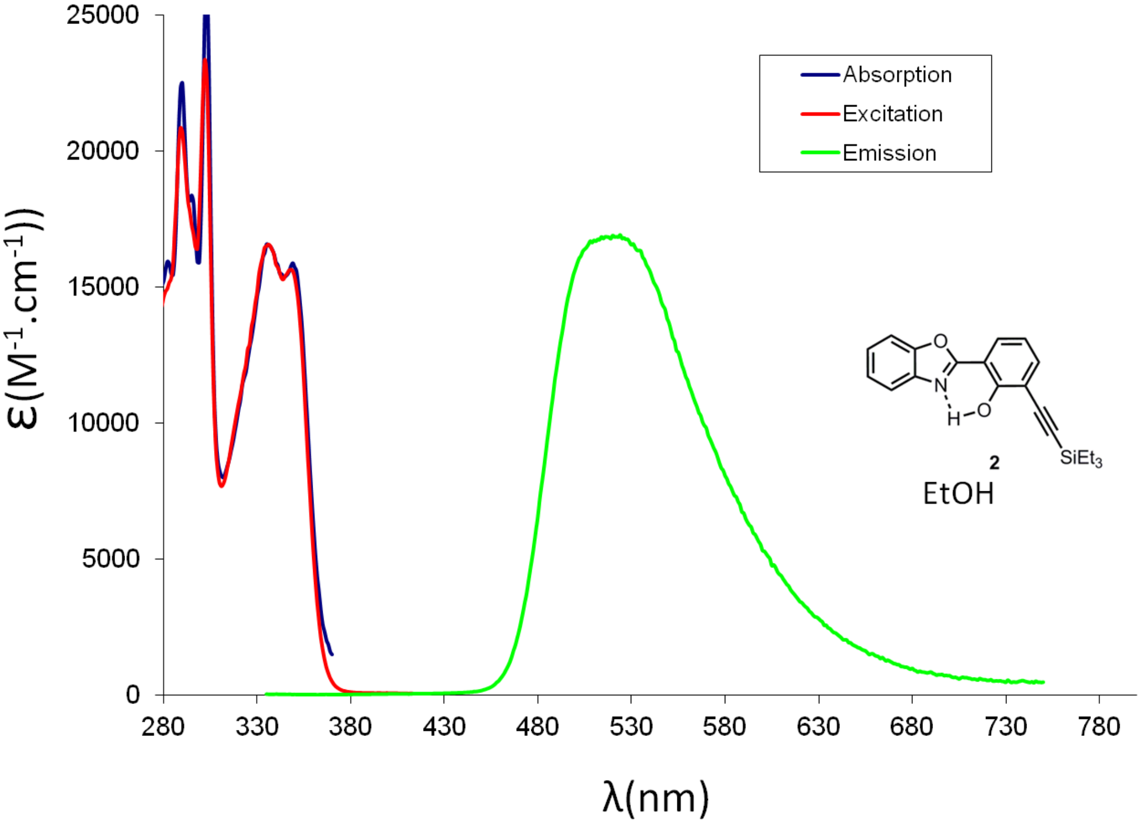
25000
20000
15000
10000
5000
0
ε(M-1.cm-1))
280
330
380
430
480
530
580
630
680
730
780
λ(nm)
Absorption
Excitation
Emission
O
N
H
O
2
SiEt3
EtOH

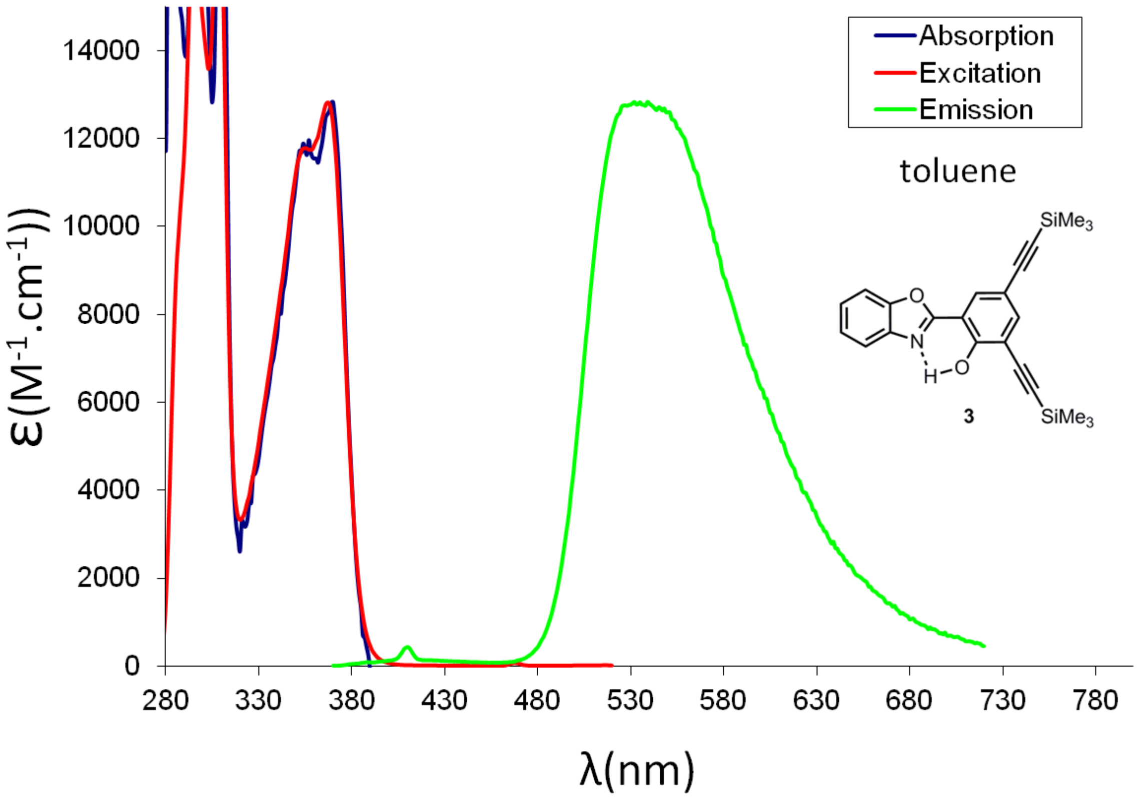

Absorption
Excitation
Emission
toluene
SiMe3
O
N
H
O
3
SiMe3
ε(M-1.cm-1))
14000
12000
10000
8000
6000
4000
2000
0
280
330
380
430
480
530
580
630
680
730
780
λ(nm)

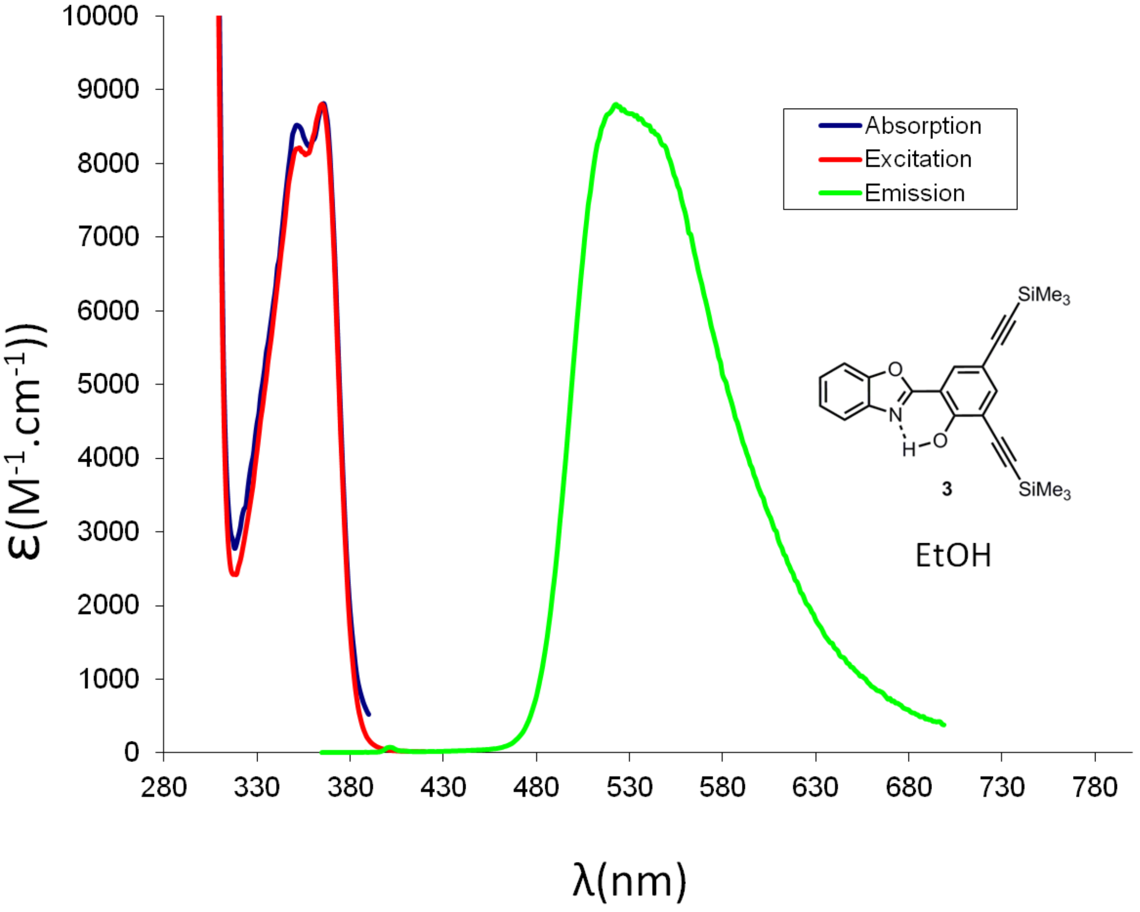

Absorption
Excitation
Emission
SiMe3
O
N
H
O
3
SiMe3
EtOH
ε(M-1.cm-1))
10000
9000
8000
7000
6000
5000
4000
3000
2000
1000
0
280
330
380
430
480
530
580
630
680
730
780
λ(nm)

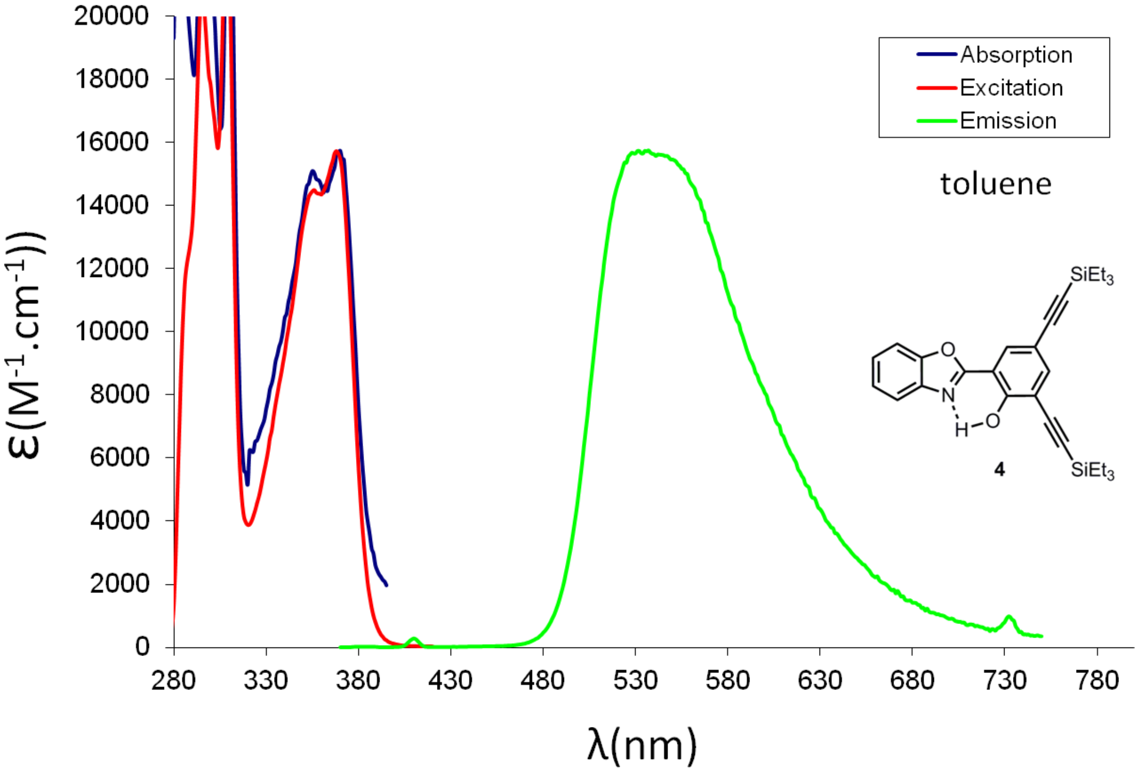
20000
18000
16000
14000
12000
10000
8000
6000
4000
2000
0
ε(M-1.cm-1))
280
330
380
430
480
530
580
630
680
730
780
λ(nm)
Absorption
Excitation
Emission
toluene
SiEt3
O
N
H
O
4
SiEt3

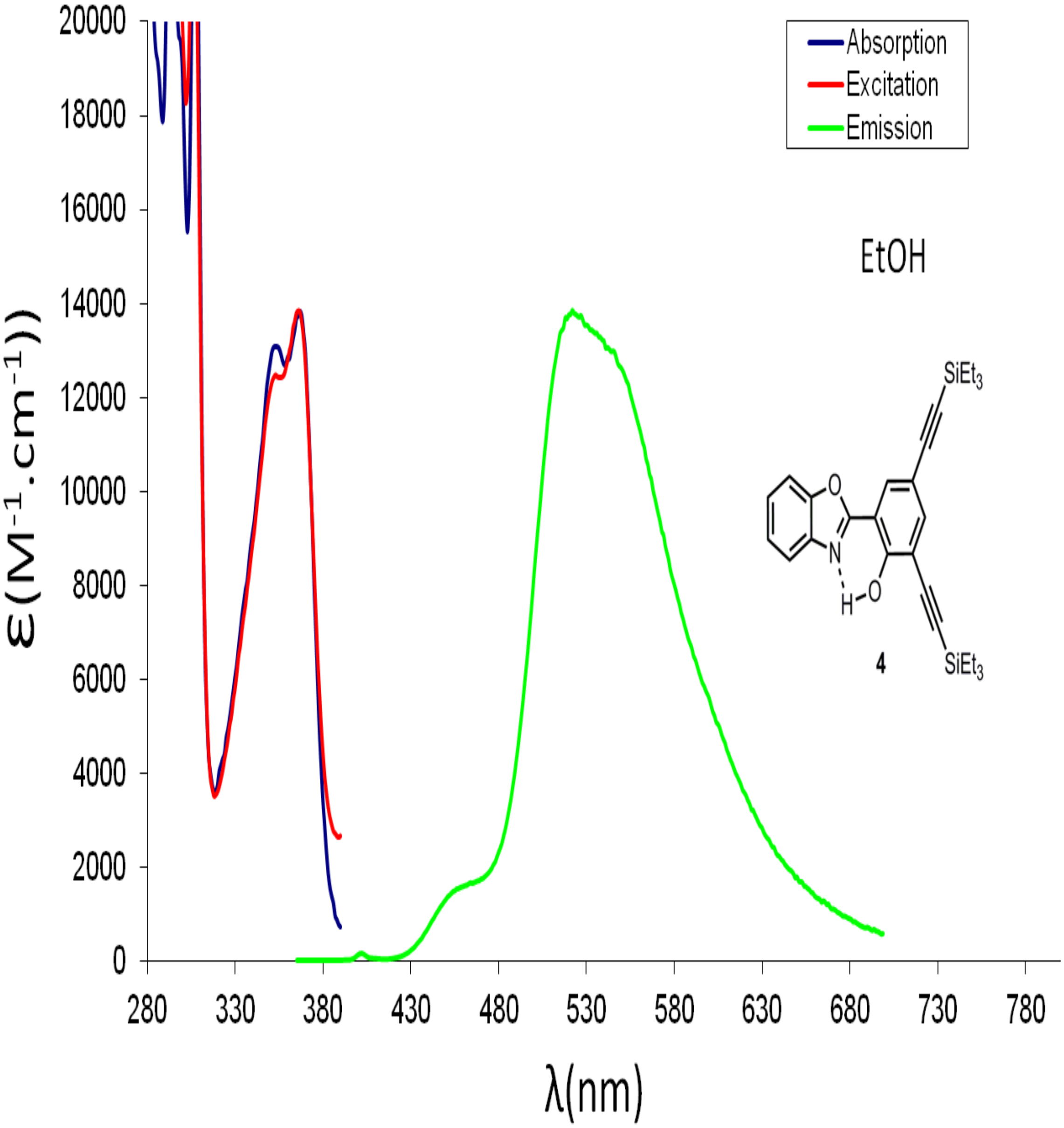
20000
18000
16000
14000
12000
10000
8000
6000
4000
2000
0
ε(M-1.cm-1))
280
330
380
430
480
530
580
630
680
730
780
λ(nm)
Absorption
Excitation
Emission
EtOH
SiEt3
O
N
H
O
4
SiEt3

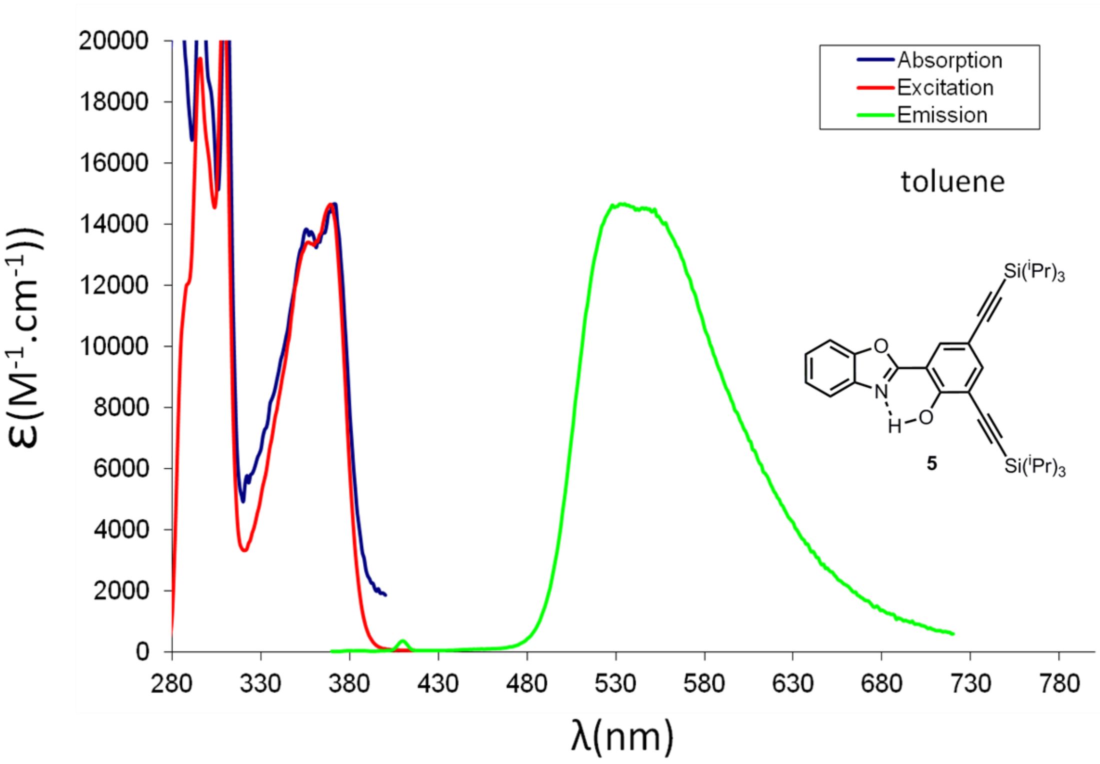

Absorption
Excitation
Emission
toluene
Si(^{i}Pr)$_3$
O
N
H
O
5
Si(^{i}Pr)$_3$
ε(M^{-1}.cm^{-1}))
λ(nm)

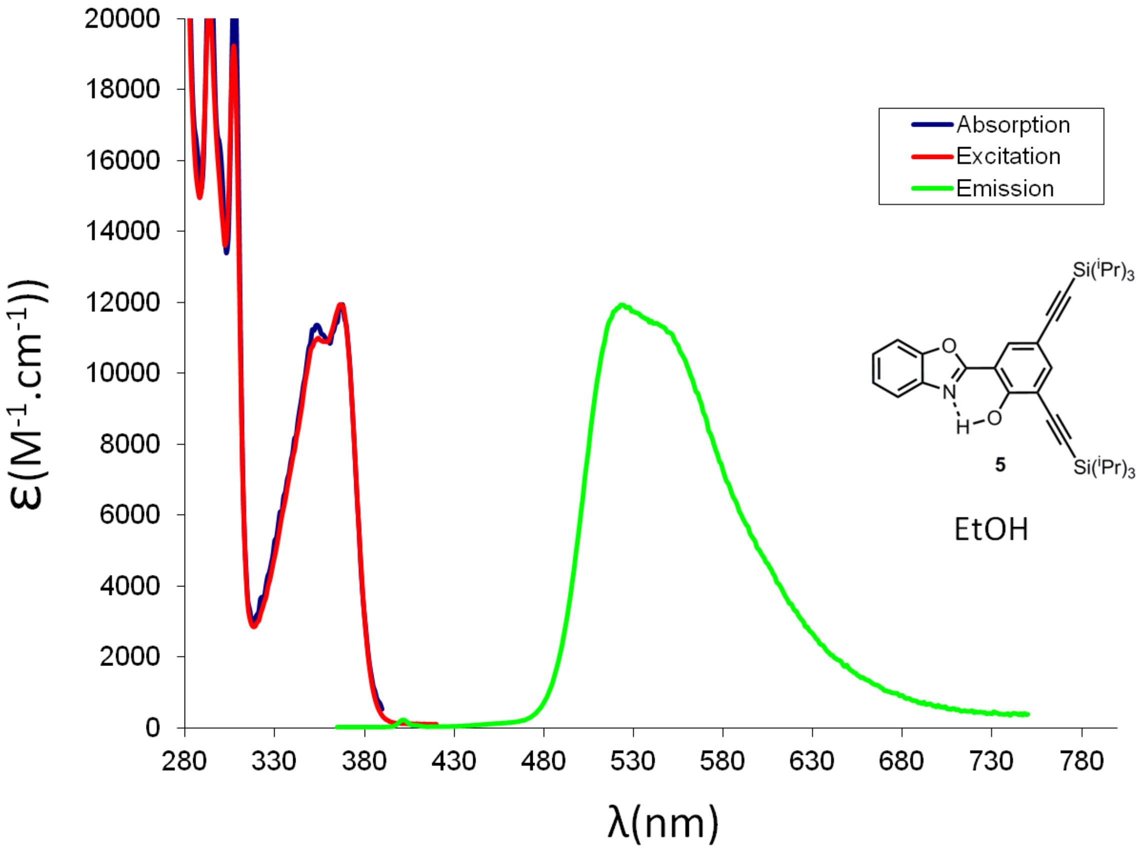

Absorption
Excitation
Emission
Si(^{i}Pr)$_3$
O
N
H
O
5
Si(^{i}Pr)$_3$
EtOH
ε(M^{-1}.cm^{-1}))
λ(nm)

S5.2 Excitation (red) and emission (green) spectra of HBO dyes 1-5 in KBr pellets

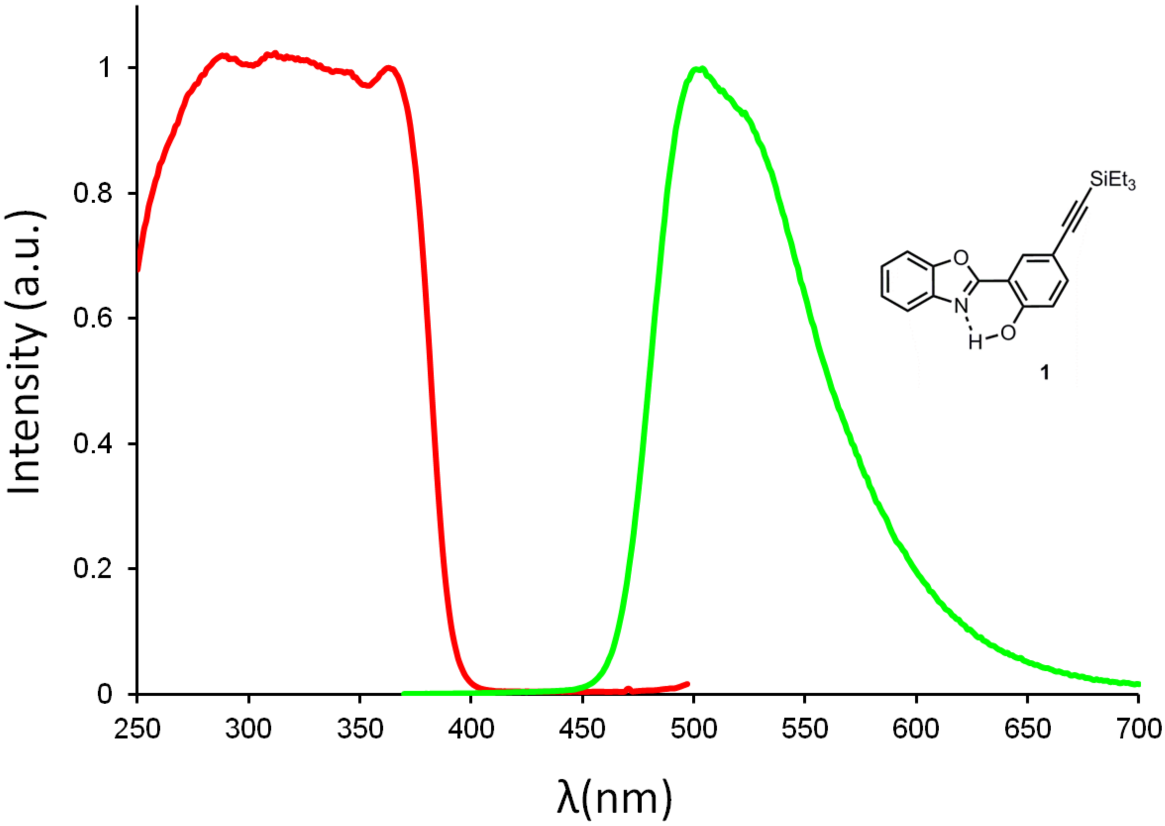

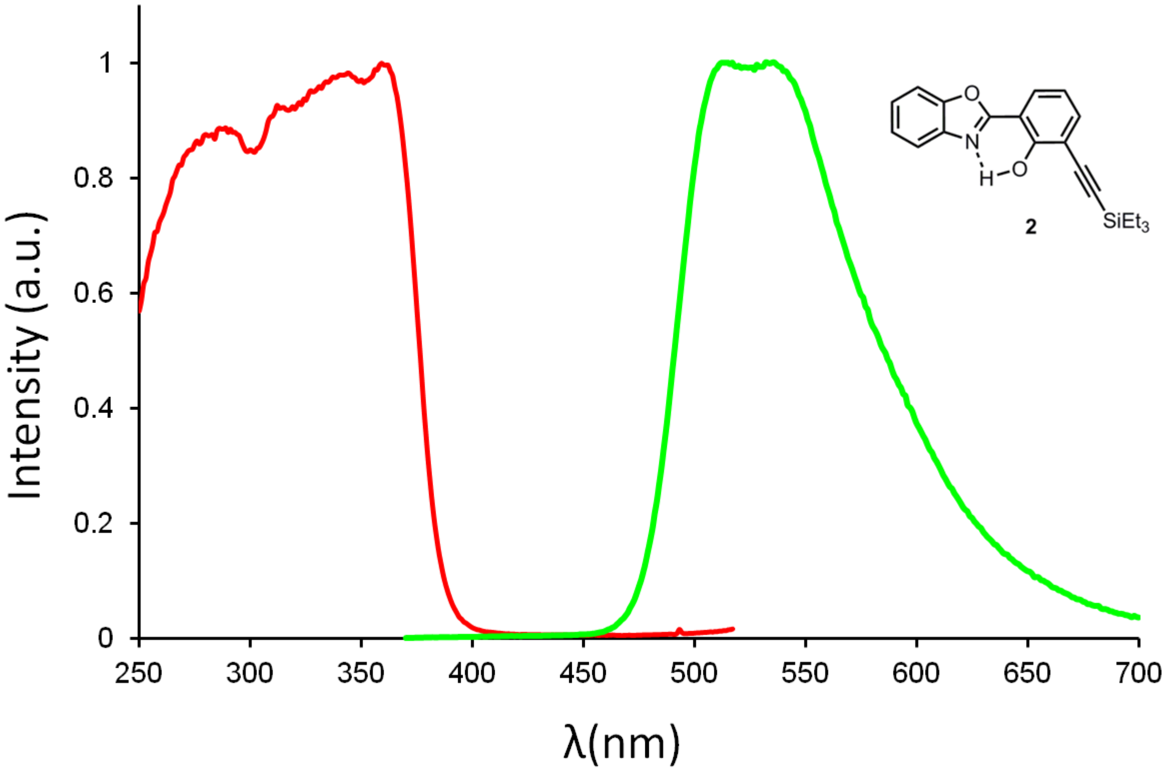

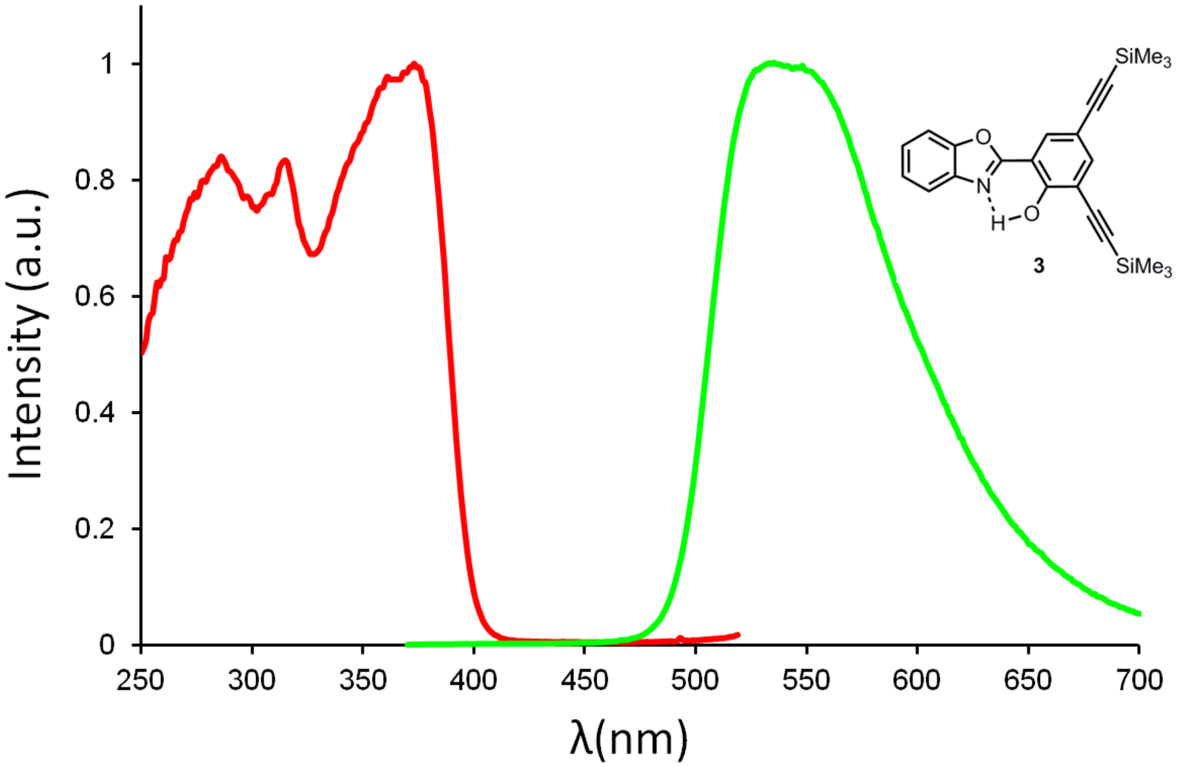
Intensity (a.u.)
λ(nm)
SiMe3
SiMe3
3

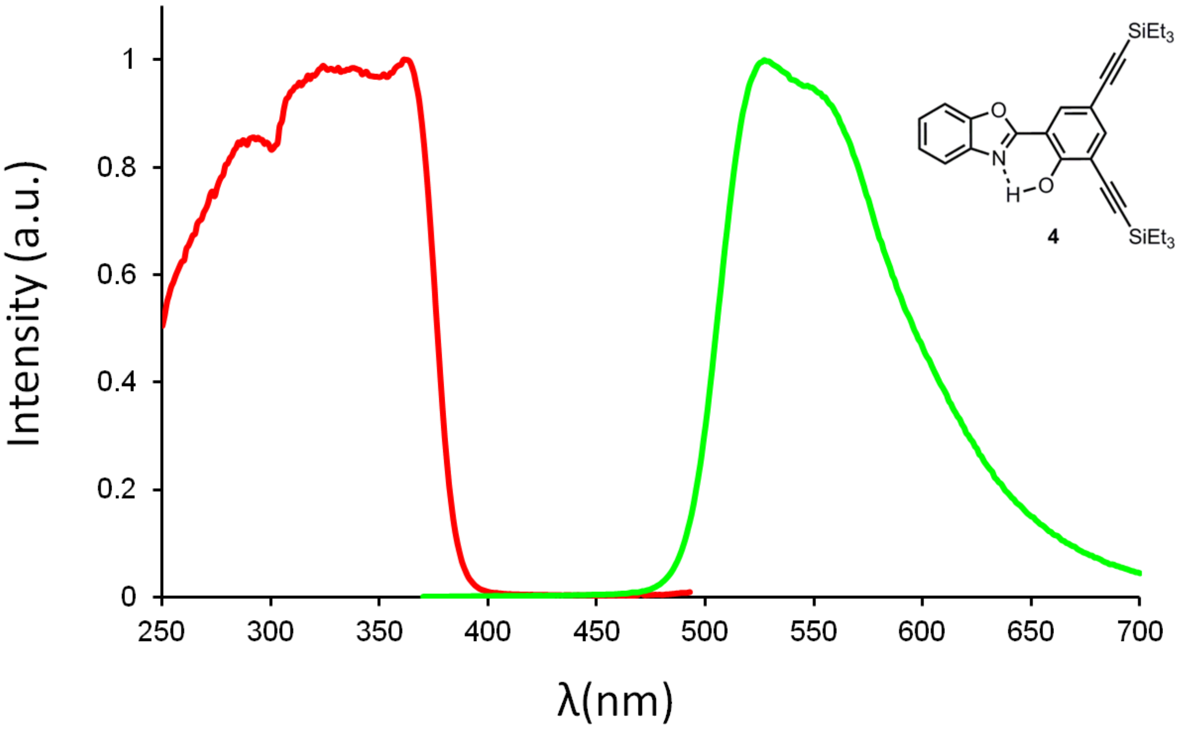
Intensity (a.u.)
λ(nm)
SiEt3
SiEt3
4

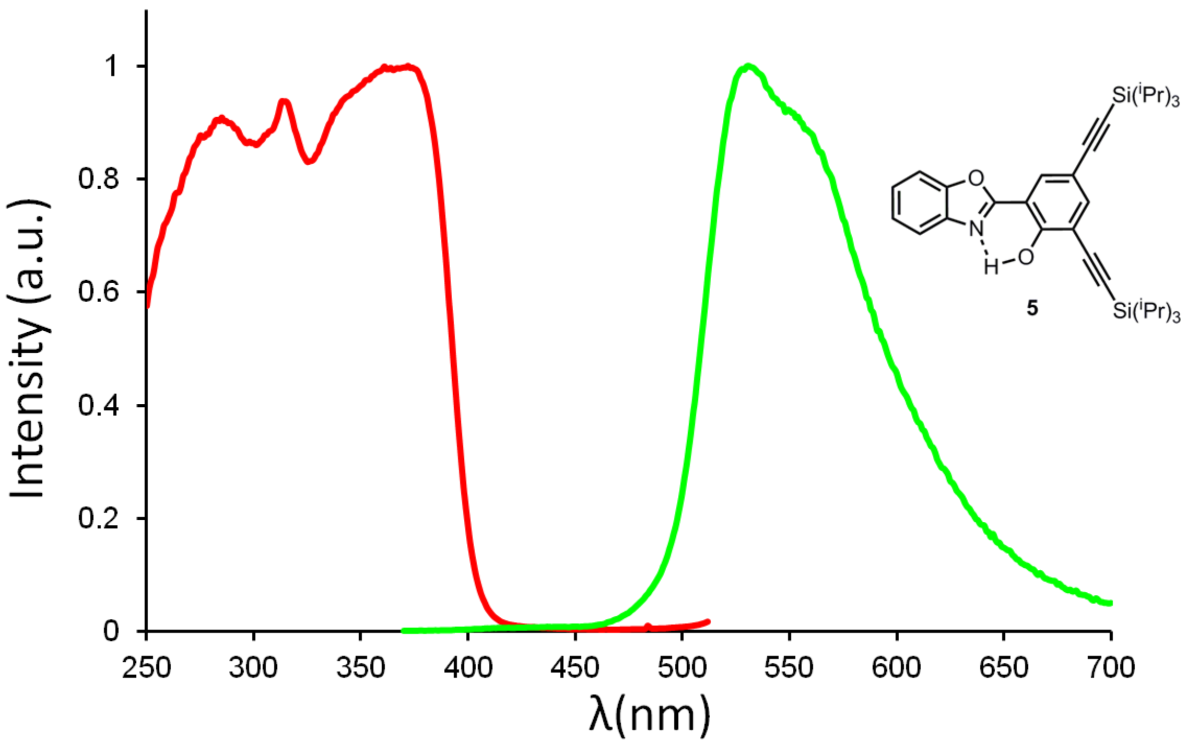
Intensity (a.u.)
1
0.8
0.6
0.4
0.2
0
250
300
350
400
450
500
550
600
650
700
λ(nm)
Si(iPr)3
O
N
H
O
5
Si(iPr)3

S5.3 Absorption, excitation and emission spectra of HBO dyes 1-5 in PMMA films

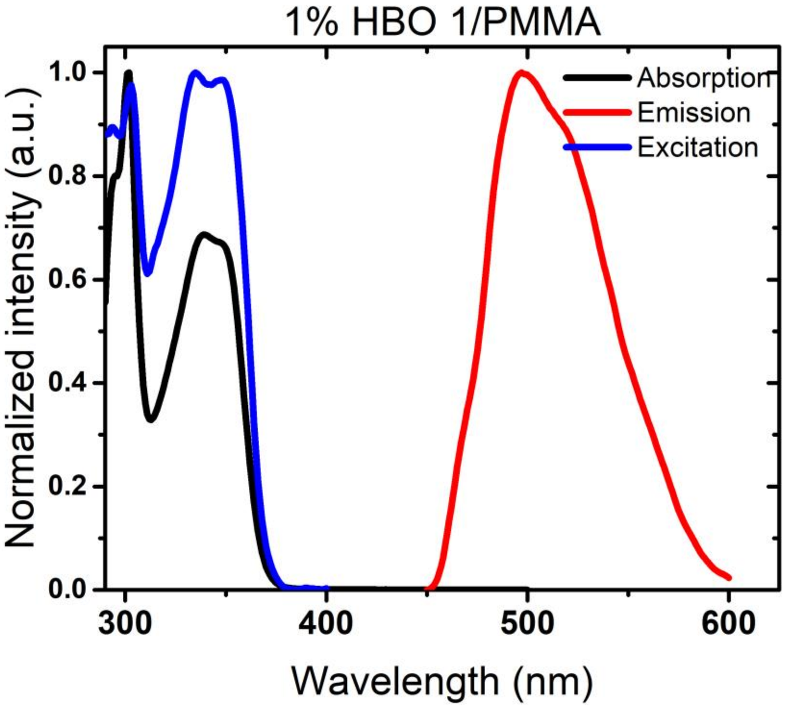

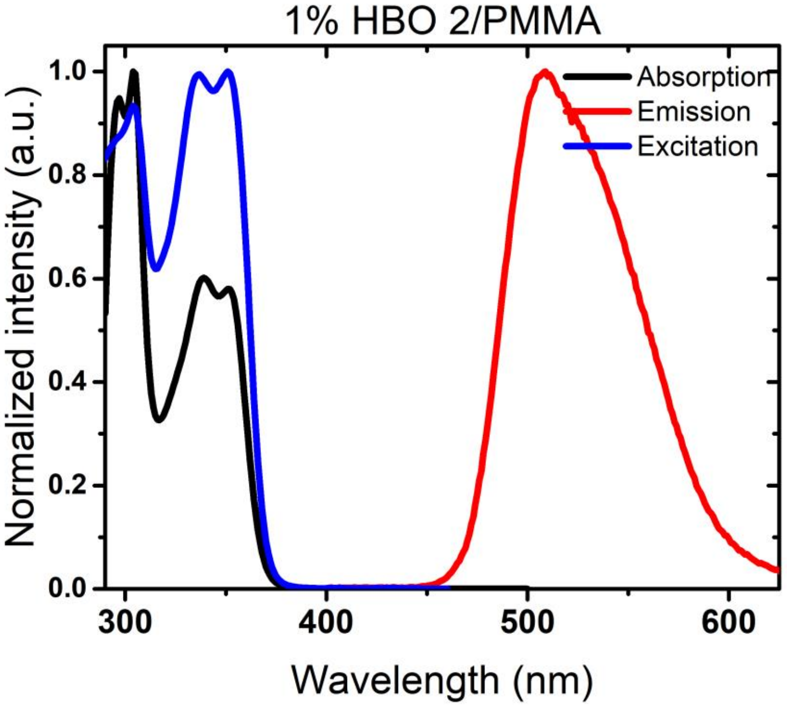

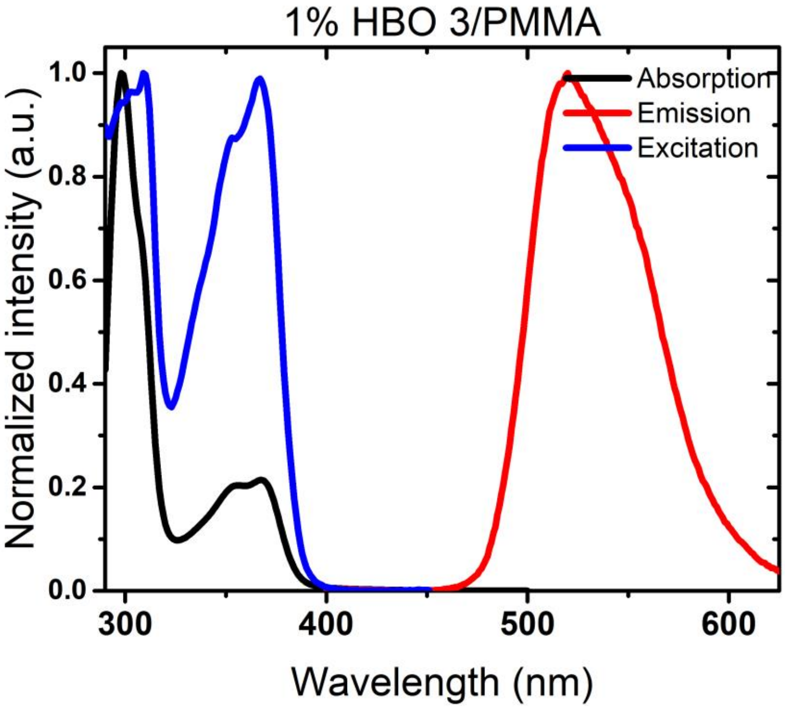
1% HBO 3/PMMA
Absorption
Emission
Excitation
Normalized intensity (a.u.)
Wavelength (nm)
0.0
0.2
0.4
0.6
0.8
1.0
300
400
500
600

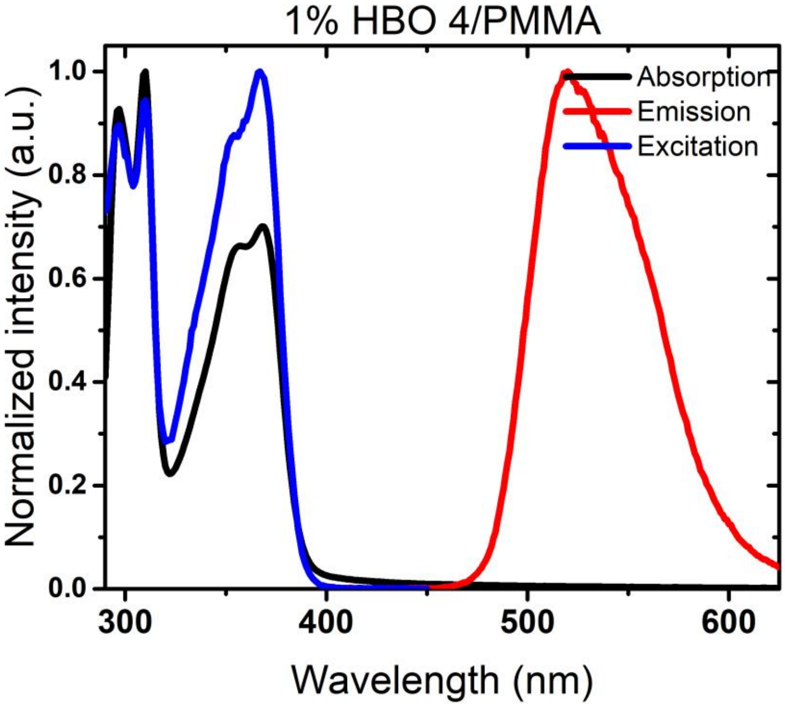
1% HBO 4/PMMA
Absorption
Emission
Excitation
Normalized intensity (a.u.)
Wavelength (nm)
0.0
0.2
0.4
0.6
0.8
1.0
300
400
500
600

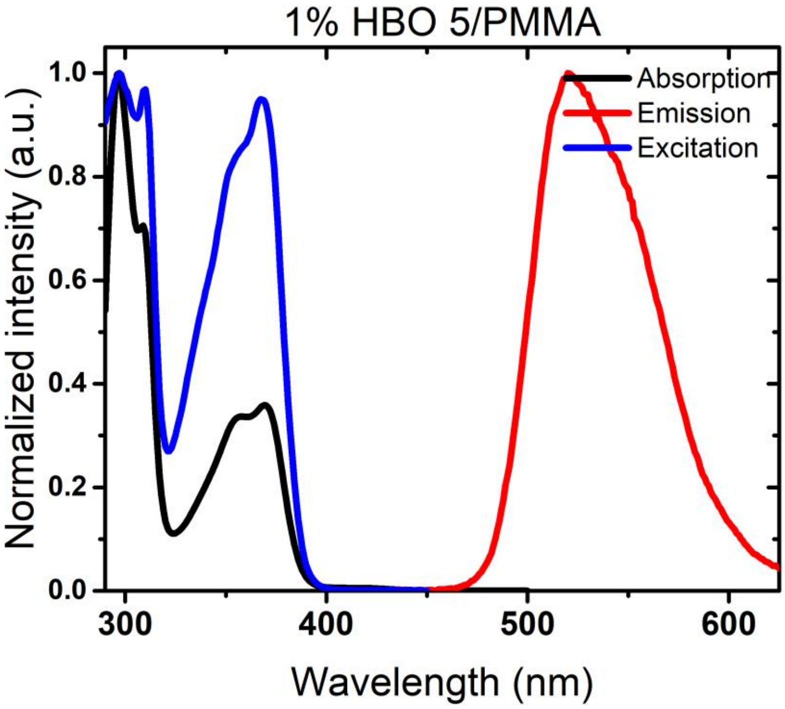

S6 Theoretical calculations

S6.1 Methods

Our computational protocol is based on current state-of-the-art for modeling ESIPT-type reactions as well as the emission and absorption spectra of comparable types of molecules.[3] We used a composite approach combining the results of Time-Dependent Density Functional Theory (TD-DFT) and the second-order algebraic diagrammatic construction [ADC(2)] method and investigated the structures as represented on the Scheme on the following page. In this composite approach, detailed in Ref. 3b, the structures and vibrations are determined by using (TD-)DFT geometries obtained with the M06-2X functional, the solvent effects are modeled with the polarizable-continuum model (PCM, here with toluene as solvent)[4] and the total and transition energies are corrected with ADC(2) to obtain theoretical best estimates. The ADC(2) calculations relied on the Resolution-of-Identity (RI) technique and were performed with default parameters. All DFT/TD-DFT calculations were performed by using Gaussian 16 software,[5] whereas all ADC(2) calculations were performed by using Turbomole 6.6.[6] For geometry optimizations, the compact 6-31G(d) atomic basis set has been used,

whereas the extended 6-311+G(2d,p) [*aug*-cc-pVTZ] atomic basis set has been applied for TD-DFT [ADC(2)] transition-energy calculations. The optimized structure of ground and excited states were confirmed by frequency calculations by using analytical Hessian for both the ground and the excited states, which resulted in no imaginary frequency for E, E* and K*, and one imaginary frequency for the transition-state structure corresponding to the proton-transfer (TS*) and K* form twist to the conical intersection (TS2*). To achieve numerical stability in the results, we used a tightened self-consistent field (10^{-10} a.u.) and geometry optimization (10^{-5} a.u.) convergence criteria and the so-called *ultrafine* (99,550) DFT-pruned integration grid during all our TD-DFT calculations. Geometry optimizations and Hessian calculations of the excited states took advantage of the linear-response (LR)[7] PCM scheme whereas for transition energies the more elaborate corrected LR scheme (cLR)[8] was used to take into account the change in the cavity polarization upon electron excitation by calculation of excited-state one-electron density. During gradient and Hessian TD-DFT calculations, we applied the *equilibrium* regime of PCM solvation (slow processes), absorption and fluorescence were treated as fast *nonequilibrium* processes. The density difference plots ($\Delta\varrho$) were obtained from the difference in the total density of the excited state and the ground state (LR-PCM-TD-DFT calculation), with the former calculated by using so-called Z-vector approach. A contour threshold of 0.008 au was used for the representation.

S6.2 Representation of a typical energy landscape

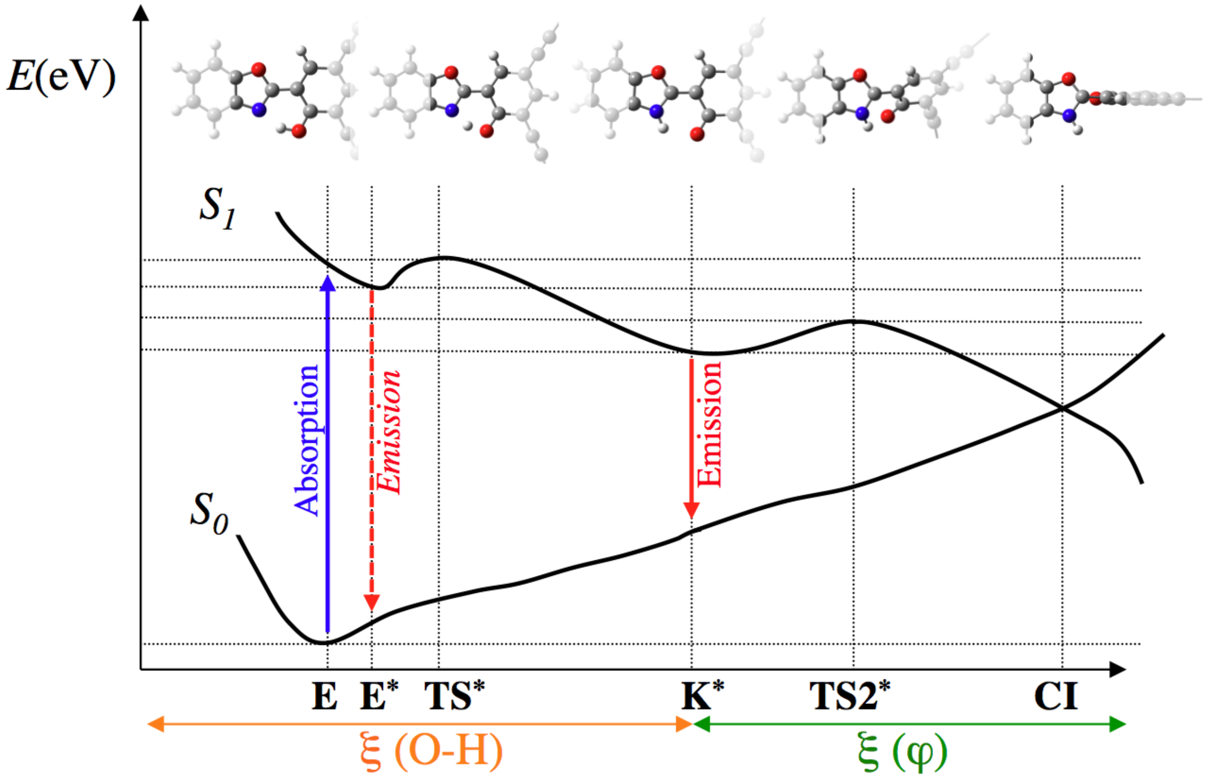

Scheme S6.1. Representation of the potential energy surface during the ESIPT process, with first a proton transfer (O-H reaction coordinate) and next a twist of the structure leading to a conical intersection (CI), back to the ground-state.

S6.3 Density difference plots

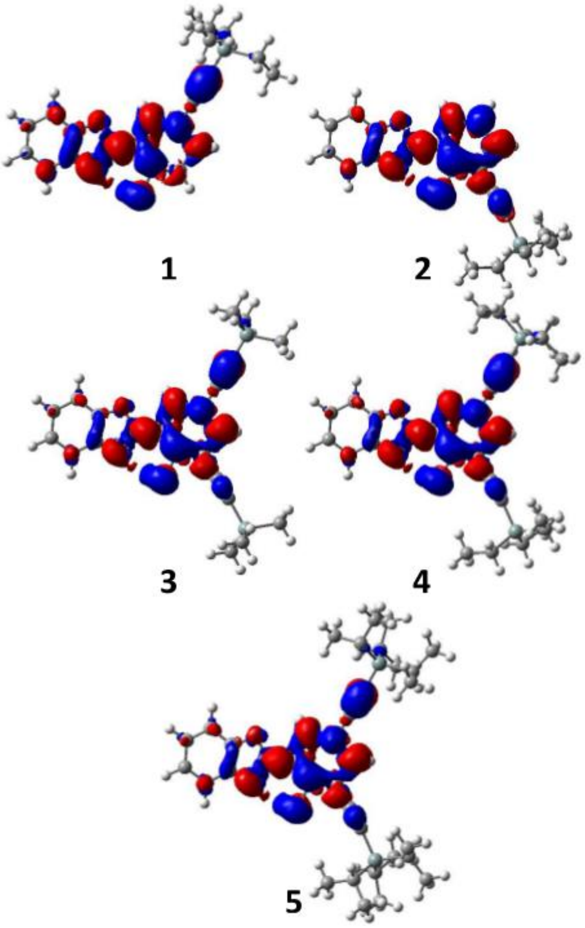

Figure S6.1. Density difference plot corresponding to electronic excitation of HBO **1-5**. The blue and red regions respectively correspond to decrease and increase of electronic density upon absorption.

S7 RL Studies

SAMPLE PREPARATION & METHODS FOR RANDOM LASING STUDIES & PHOTODEGRADATION

A series of polymeric thin films doped with HBO dyes were prepared by drop casting method as described elsewhere.19 Samples were made according to following procedure: commercially available PMMA in powder form (M_w = 350 kDa, Sigma Aldrich®) was added to toluene (POCH S.A.) with concentration of 5% w/w (dry weight proportion). This solution was stirred and left for complete polymer dissolution. A separate solutions of HBO dyes in toluene were made with 1% w/w, then mixed with PMMA solution with concentration varying from 1% to 5% w/w (with respect to PMMA). The final solution was stirred for 24h for stabilization preceding deposition on the glass plate by the drop casting technique. Polymeric ESIPT doped films were left in solvent saturated atmosphere for 48h at room temperature for toluene evaporation. For random lasing experiments, the triplet in frequency Nd:YAG nanosecond laser beam (355 nm, 6 ns) was directed through a half-wave plate, polarizer and series of convex and cylindrical lenses incident onto the sample surface, thus obtaining stripe shaped area of illumination with uniform intensity in dimension of 5 mm x 0.5 mm. Emitted light coming from the sample was collected perpendicularly to the investigated layer surface plane by a high resolution Andor Shamrock SR-163 fiber spectrometer in a function of excitation energy density.

For photodegradation measurements we used following definition: a number of pulses upon which the intensity of emission drops by half ($t_{1/2}$). Setup was the same, as for RL studies described previously, alas intensity of input power was constant (2mJ cm^{-2}).

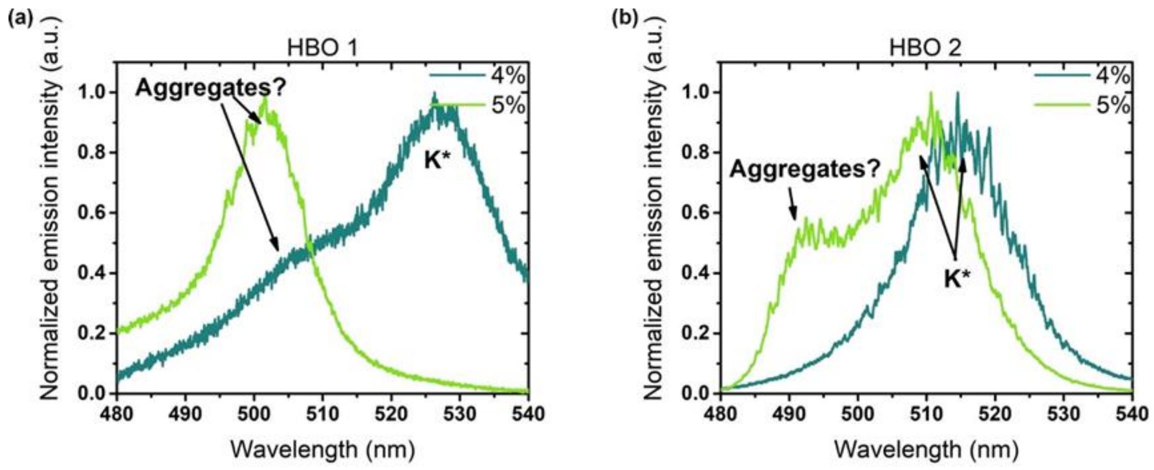

Figure S7.1 Comparison of emission profile of 4% and 5% HBO **1** (a) and HBO **2** (b) in PMMA matrix. For HBO **1** blue-shifted emission from the aggregated form is visible, whereas for HBO **2** dual emission is observed.

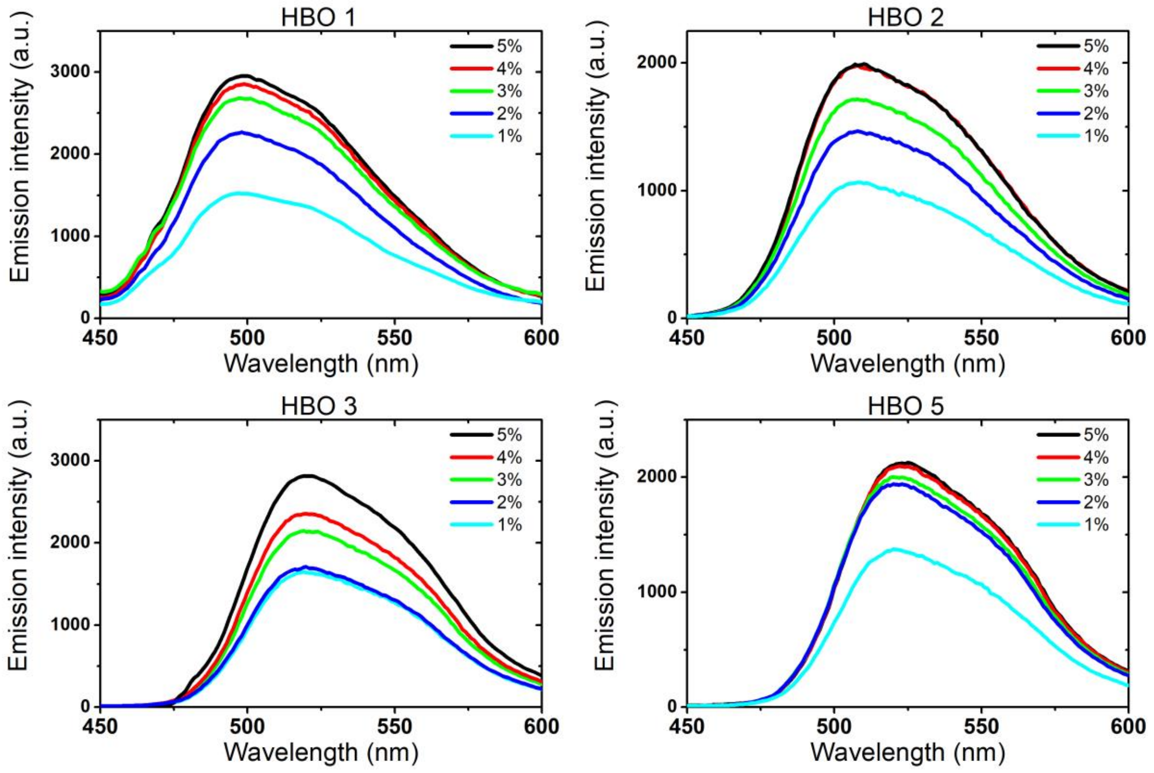

Figure S7.2 Comparison of emission profiles of 1-5% HBO dyes in PMMA matrix.

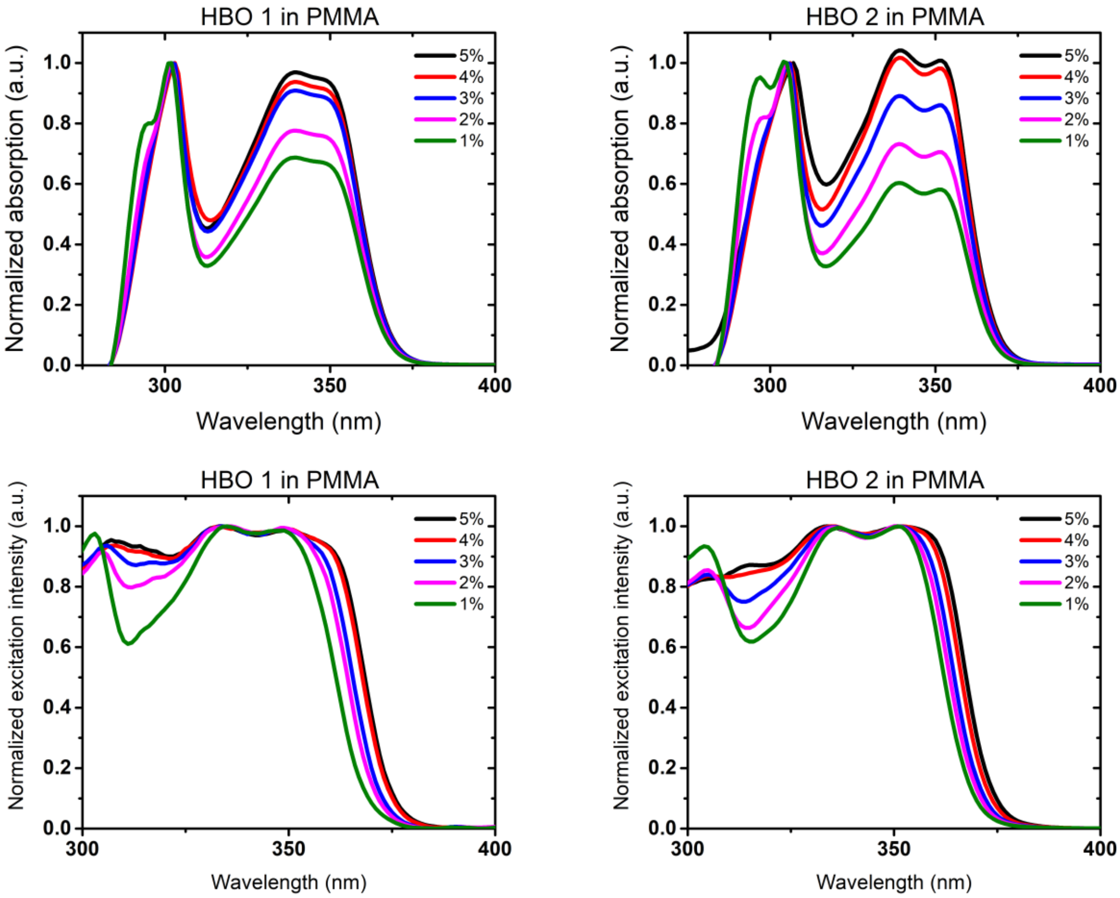

Figure S7.3 Spectroscopic measurements of HBO 1 and 2 doped PMMA films.

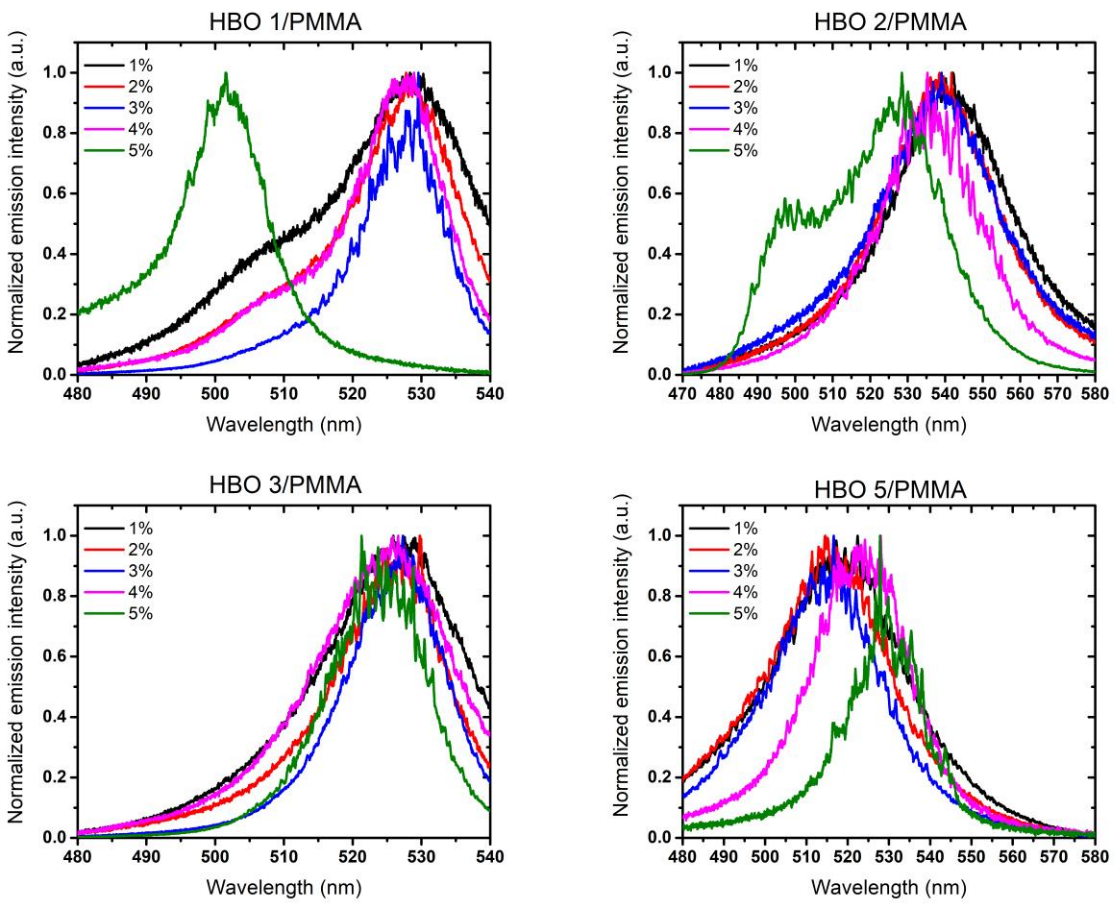

Figure S7.4 Dependence of the light emission spectra on the doping concentration of ESIPT dyes 1-3 and 5 in the PMMA matrix.

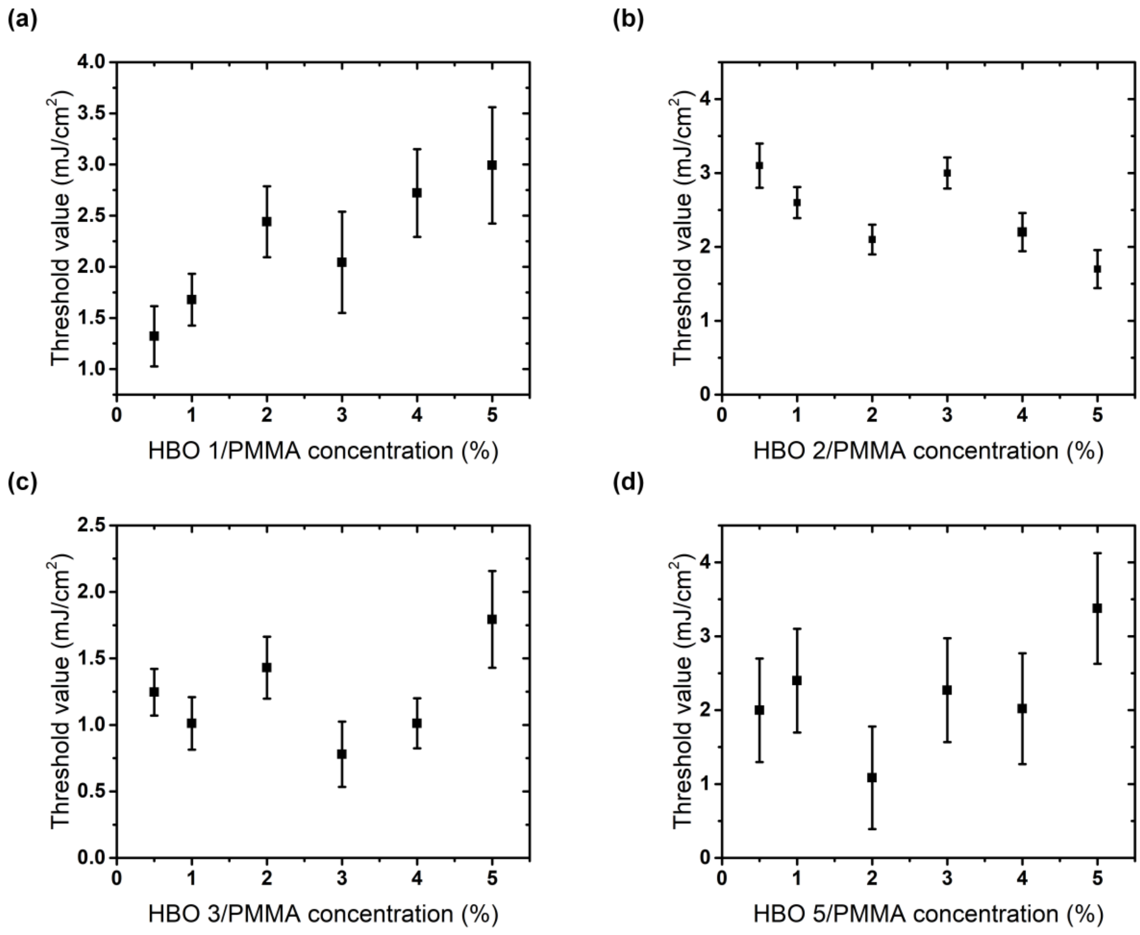

Figure S7.5 Dye concentration influence on RL threshold values for HBO/PMMA thin films. The error bars are noted on the graphs.

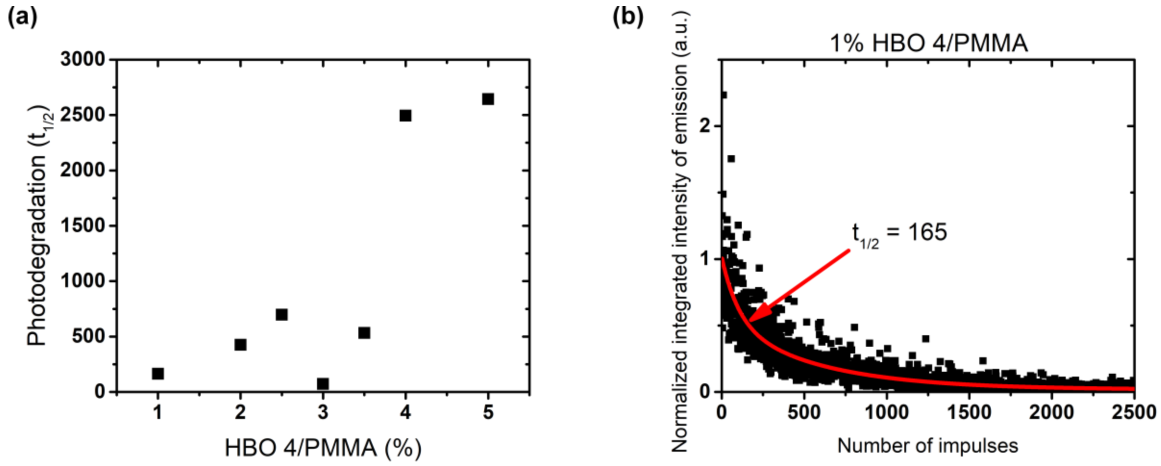

Figure S7.6 (a) Photodegardation results and (b) influence of number of pulses on emission intensity of 1% HBO 4/PMMA thin films.

Table S7.1. Comparison of random lasing properties for dyes **1-5** in PMMA matrix. We report the maximum emission wavelengths of the keto forms (dyes **1-5**) and of the aggregated forms (dyes **1, 2**), in nm.

Dye	E density at threshold value (mJ/cm^2)	λ_{em},agg (nm)	λ_{em} K* (nm)
**1**	1.7	500	525
**2**	2.6	495	540
**3**	1.0	-	525
**4**	0.5	-	555
**5**	1.4	-	525

References

1. J. Massue, G. Ulrich, R. Ziessel, *Eur. J. Org. Chem.*, **2013**, 25, 5701–5709.
2. J. Massue, D. Frath, P. Retailleau, G. Ulrich, R. Ziessel, *Chem. Eur. J.*, **2013**, 19, 5375–5386.
3. (a) M. Raoui, J. Massue, C. Azarias, D. Jacquemin, G. Ulrich, *Chem. Commun*. **2016**, 52, 9216–9219; (b) C. Azarias, S. Budzak, A. D. Laurent, G. Ulrich, D. Jacquemin, *Chem. Sci*. **2016**, 7, 3763–3774; (c) Y. Houari, S. Chibani, D. Jacquemin, A. D. Laurent, *J. Phys. Chem. B* **2015**, 119, 2180–2192; (d) M. Savarese, P. A. Netti, C. Adamo, N. Rega, I. Ciofini, *J. Phys. Chem. B* **2013**, 117, 16165–16173; (e) L. Wilbraham, M. Savarese, N. Rega, C. Adamo, I. Ciofini, *J. Phys. Chem. B* **2015**, 119, 2459–2466.
4. J. Tomasi, B. Mennucci, R. Cammi, *Chem. Rev.* **2005**, 105, 2999–3093

5. Gaussian 16, Revision A.03, M. J. Frisch, G. W. Trucks, H. B. Schlegel, G. E. Scuseria, M. A. Robb, J. R. Cheeseman, G. Scalmani, V. Barone, G. A. Petersson, H. Nakatsuji, X. Li, M. Caricato, A. V. Marenich, J. Bloino, B. G. Janesko, R. Gomperts, B. Mennucci, H. P. Hratchian, J. V. Ortiz, A. F. Izmaylov, J. L. Sonnenberg, D. Williams-Young, F. Ding, F. Lipparini, F. Egidi, J. Goings, B. Peng, A. Petrone, T. Henderson, D. Ranasinghe, V. G. Zakrzewski, J. Gao, N. Rega, G. Zheng, W. Liang, M. Hada, M. Ehara, K. Toyota, R. Fukuda, J. Hasegawa, M. Ishida, T. Nakajima, Y. Honda, O. Kitao, H. Nakai, T. Vreven, K. Throssell, J. A. Montgomery, Jr., J. E. Peralta, F. Ogliaro, M. J. Bearpark, J. J. Heyd, E. N. Brothers, K. N. Kudin, V. N. Staroverov, T. A. Keith, R. Kobayashi, J. Normand, K. Raghavachari, A. P. Rendell, J. C. Burant, S. S. Iyengar, J. Tomasi, M. Cossi, J. M. Millam, M. Klene, C. Adamo, R. Cammi, J. W. Ochterski, R. L. Martin, K. Morokuma, O. Farkas, J. B. Foresman, and D. J. Fox, Gaussian, Inc., Wallingford CT, 2016.
6. TURBOMOLE V6.6 2014, a development of University of Karlsruhe and Forschungszentrum Karlsruhe GmbH, 1989 – 2007, TURBOMOLE GmbH, since 2007; available from http://www.turbomole.com
7. R. Cammi, B. Mennucci, *J. Chem. Phys.* **1999**, 110, 9877–9886
8. (a) R. Cammi, S. Corni, B. Mennucci, J. Tomasi, J. Chem. Phys. **2005**, 122, 104513; (b) M. Caricato, B. Mennucci, J. Tomasi, F. Ingrosso, R. Cammi, S. Corni, G. Scalmani, *J. Chem. Phys*. **2006**, 124, 124520.
